# Optogenetically engineered Ca^2+^ oscillation-mediated DRP1 activation promotes mitochondrial fission and cell death

**DOI:** 10.1242/jcs.260819

**Published:** 2023-06-21

**Authors:** Yi-Shyun Lai, Cheng-Chi Chang, Yong-Yi Chen, Thi My Hang Nguyen, Jixuan Xu, Ying-Chi Chen, Yu-Fen Chang, Chia-Yih Wang, Pai-Sheng Chen, Shih-Chieh Lin, I-Chen Peng, Shaw-Jenq Tsai, Wen-Tai Chiu

**Affiliations:** ^1^ Department of Biomedical Engineering, National Cheng Kung University, Tainan 701, Taiwan; ^2^ Department of Chemistry, National Cheng Kung University, Tainan 701, Taiwan; ^3^ LumiSTAR Biotechnology, Taipei 115, Taiwan; ^4^ Department of Cell Biology and Anatomy, National Cheng Kung University, Tainan 701, Taiwan; ^5^ Department of Medical Laboratory Science and Biotechnology, National Cheng Kung University, Tainan 701, Taiwan; ^6^ Institute of Basic Medical Sciences, National Cheng Kung University, Tainan 701, Taiwan; ^7^ Department of Life Sciences, National Cheng Kung University, Tainan 701, Taiwan; ^8^ Department of Physiology, National Cheng Kung University, Tainan 701, Taiwan; ^9^ Medical Device Innovation Center, National Cheng Kung University, Tainan 701, Taiwan

**Keywords:** Ca^2+^ oscillation, DRP1, Mitochondrial fission, Optogenetics

## Abstract

Mitochondrial dynamics regulate the quality and morphology of mitochondria. Calcium (Ca^2+^) plays an important role in regulating mitochondrial function. Here, we investigated the effects of optogenetically engineered Ca^2+^ signaling on mitochondrial dynamics. More specifically, customized illumination conditions could trigger unique Ca^2+^ oscillation waves to trigger specific signaling pathways. In this study, we found that modulating Ca^2+^ oscillations by increasing the light frequency, intensity and exposure time could drive mitochondria toward the fission state, mitochondrial dysfunction, autophagy and cell death. Moreover, illumination triggered phosphorylation at the Ser616 residue but not the Ser637 residue of the mitochondrial fission protein, dynamin-related protein 1 (DRP1, encoded by *DNM1L*), via the activation of Ca^2+^-dependent kinases CaMKII, ERK and CDK1. However, optogenetically engineered Ca^2+^ signaling did not activate calcineurin phosphatase to dephosphorylate DRP1 at Ser637. In addition, light illumination had no effect on the expression levels of the mitochondrial fusion proteins mitofusin 1 (MFN1) and 2 (MFN2). Overall, this study provides an effective and innovative approach to altering Ca^2+^ signaling for controlling mitochondrial fission with a more precise resolution than pharmacological approaches in the temporal dimension.

## Teaser

**Summary:** Optogenetically driven Ca^2+^ oscillation promotes mitochondrial fission by increasing phosphorylation at Ser616 of DRP1 via the activation of CaMKII, ERK and CDK1.

## INTRODUCTION

Calcium (Ca^2+^) affects nearly all aspects of living cells and drives various physiological processes in the cell (Berridge et al., 1998; Pinton et al., 2008). Ca^2+^-mediated cell signaling is regulated by channels and transporters in the plasma membrane, endoplasmic reticulum (ER) and mitochondria, which control the concentration and balance of cytosolic Ca^2+^ to achieve different spatial and temporal dynamics (Berridge et al., 2003). Intracellular Ca^2+^ is precisely controlled considering its essential role in cellular physiology. Ca^2+^ oscillation is a pervasive signal present in all cells, which provides an effective way to transmit intracellular biological information; for example, Ca^2+^ oscillation controls the release of neurotransmitters in neuron cells (Fu et al., 2008), the contraction of smooth muscle cells in pulmonary and vascular vessels (Pauvert et al., 2000; Perez and Sanderson, 2005; Fan et al., 2007), gene expression and cell differentiation (Yildirim and Bertram, 2017; Okada et al., 2020). Ca^2+^ oscillation has been reported to be related to the activation of intracellular signaling, such as the regulation of intracellular transcription factors (Lai et al., 2021) and mitochondrial metabolism (Wacquier et al., 2016). Moreover, a number of direct and indirect mechanisms regulate the amplitude, frequency and duty cycle of intracellular Ca^2+^ oscillations (Parekh, 2011; Smedler and Uhlén, 2014). In addition, Ca^2+^ controls the quality of cellular organelles; a loss of balance in Ca^2+^ trafficking might lead to reactive oxygen species (ROS) accumulation in the endoplasmic reticulum and mitochondria, leading to cellular apoptosis and autophagy (Kaufman and Malhotra, 2014). In addition to supplying cellular energy, mitochondria also participate in Ca^2+^ signaling. Ca^2+^ sequestration in the mitochondria can regulate the rate of oxidative metabolism and is important for the control of cellular ATP homeostasis (Boyman et al., 2020). Mitochondria can facilitate the storage of Ca^2+^ and buffer cytosolic Ca^2+^ concentration to regulate the cell fate. For example, Ca^2+^ overload might activate cellular apoptosis or necrosis by causing transient opening of a channel on the mitochondrial inner membrane or the permeability transition pore with an increase in mitochondrial permeability (Gunter et al., 2004). In contrast, low Ca^2+^ levels might promote pro-survival cellular processes and alleviate ATP production (Rizzuto et al., 2012).

Mitochondrial quality is a critical determinant of cellular survival because unbalanced Ca^2+^ levels can lead to programmed cell death. Moreover, several neurodegenerative diseases, including Parkinson's, Alzheimer's and Huntington's diseases, are related to mitochondrial quality control (Chen and Chan, 2009). Mitochondrial quality is regulated by its fusion and fission machinery, a process also known as mitochondrial dynamics, which is important for the maintenance of mitochondrial function and is a constituent of cellular quality control. Mitochondrial fusion enhances mitochondrial integrity and is a cardinal process for maintaining cell health. Membrane fusion of separated mitochondria is achieved by membrane fusion proteins, such as *N*-ethylmaleimide-sensitive factors (NSFs) and soluble NSF-attachment protein receptors, which help merge the lipid bilayers of the outer membrane (Choi et al., 2006). As mitochondria are double membrane-bounded organelles, the conserved mitofusin (MFN) protein isoforms in mammals, MFN1 and MFN2, at the mitochondrial outer membrane and OPA1 at the inner membrane cooperate to induce mitochondrial fusion by tethering the outer and inner mitochondrial membranes (Westermann, 2010). Most cellular pathological events are associated with a change toward reduced fusion in the mitochondrial equilibrium. Proteins, such as PTEN-induced kinase 1, parkin, and Parkinsonism-associated deglycase, promote mitochondrial fission and/or inhibit fusion by negatively regulating fusion protein function. These modifications in fusion and fission might represent a novel therapeutic strategy for Parkinson's disease (Deng et al., 2008). Mitochondrial fission separates damaged mitochondria to stabilize the health of the mitochondrial network, thereby regulating mitochondrial dynamics and promoting mitochondrial trafficking (Bossy-Wetzel et al., 2003). Dynamin-related protein 1 (DRP1) is gathered from the cytosol and assembled into multimeric spirals that wrap around the mitochondria to facilitate the separation of the inner and outer membranes (Youle and van der Bliek, 2012). Mitochondrial fission is also related to cellular events, such as apoptosis, and the mitochondrial fission machinery actively participates in the process of programmed cell death (Youle and Karbowski, 2005). In contrast, mitochondrial dysfunction can also result in a reduction in cellular ATP levels, inhibition of cell proliferation and increase in autophagy (Parone et al., 2008). Thus, the monitoring and regulation of mitochondrial fission are critically important for the maintenance of overall cell health.

As a ubiquitous secondary messenger, Ca^2+^ affects numerous cellular pathways, such as the downstream protein kinase C, Ca^2+^/calmodulin-dependent protein kinase (CAMK), extracellular signal-regulated protein kinases 1 and 2 (encoded by *MAPK3* and *MAPK1*, respectively; collectively referred to as ERK1/2), and calcineurin pathways (Hama et al., 1995; Agell et al., 2002; Mohapatra and Nau, 2005). Moreover, these Ca^2+^-related signaling pathways also affect mitochondrial dynamics via downstream molecular pathways, eventually leading to disturbances in the cell fate (Youle and Karbowski, 2005; Parone et al., 2008). Ca^2+^ regulates morphological changes in the mitochondria. For example, cytosolic Ca^2+^ elevation can promote mitochondrial fission via the activation of phosphatase calcineurin and dephosphorylation of downstream DRP1 at serine 637 (S637) (Cribbs and Strack, 2007). A sustained increase in cytosolic Ca^2+^ levels can activate the interaction between cytosolic calcineurin and DRP1. Calcineurin-dependent dephosphorylation is targeted at the conserved S637 residue of DRP1. Subsequently, the pro-fission protein DRP1 is translocated to mitochondria to induce mitochondrial fragmentation, as substantiated by site-directed mutagenesis (Cereghetti et al., 2008). Thus, calcineurin-dependent mitochondrial fission is dependent on a process involving a sustained increase in Ca^2+^ levels, activation of calcineurin and dephosphorylation of DRP1 at the S637 residue, followed by its translocation to the mitochondria. Moreover, CAMKII regulates and increases the phosphorylation of DRP1 at the serine 616 (S616) residue (Bo et al., 2018), and a transient increase in Ca^2+^ levels activates ERK1/2 signaling and facilitates mitochondrial fragmentation via DRP1-S616 phosphorylation (Yu et al., 2011). Moreover, activation of ERK-promoted mitochondrial fission also affects neuronal diseases, such as Huntington's disease, via phosphorylation of DRP1-S616 (Roe and Qi, 2018). During mitosis, cyclin-dependent kinase 1 (CDK1) phosphorylates DRP1 at S616 to induce mitochondrial fission and cell reprogramming (Wang et al., 2014). Mitotic phosphorylation of DRP1 is induced by CDK1, which facilitates mitosis and is suggested to contribute to mitochondrial segregation in cycling cells (Zunino et al., 2009). In conclusion, elevation of cytosolic Ca^2+^ can promote DRP1 recruitment and activate its GTPase activity to further induce mitochondrial fission (Chang and Blackstone, 2007; Sabouny and Shutt, 2020).

Optogenetics combines genetic and optical principles to achieve precise control of cellular functions (Deisseroth, 2011). Appropriate light-activated proteins can be expressed in specific regions to function as light-gated ion channels (Wong et al., 2012). Using temporally and spatially controlled light stimulation, cell activities affected by these light-sensitive proteins can be precisely controlled (Pastrana, 2011). Channel-rhodopsin 2 (ChR2) is a direct light-switched cation-selective ion channel that was first identified as a microbial-type rhodopsin native to the green alga *Chlamydomonas reinhardtii*. Ca^2+^-translocating channel-rhodopsin (CatCh), an L132C mutant of wild-type ChR2, is the preferred tool for both *in vivo* and *in vitro* observations of Ca^2+^ oscillations (Nagel et al., 2003). CatCh can provide a voltage response with a full response time and ∼70-fold greater light sensitivity than that of wild-type ChR2. CatCh also shows higher affinity and permeability to Ca^2+^ than to Na^+^ in comparison with those of wild-type ChR2 (Kleinlogel et al., 2011). Optogenetic manipulation can actively control Ca^2+^ signals in ways that cannot be achieved by pharmacological methods, which include frequency, amplitude, duty cycle and work duration. Through pharmacological approaches, Ca^2+^ signals can only be recorded passively (Carter and de Lecea, 2011; Lai et al., 2021). Ca^2+^ and its downstream signaling pathways mediate mitochondrial dynamics by modifying DRP1 phosphorylation at S616 or dephosphorylation at S637, both of which can facilitate mitochondrial fission. This study aimed to explore the regulation of mitochondrial dynamics via the precise control of light-generated Ca^2+^ oscillations.

## RESULTS

### Ca^2+^-mediated mitochondrial morphology transitions

**Fig. 1.**
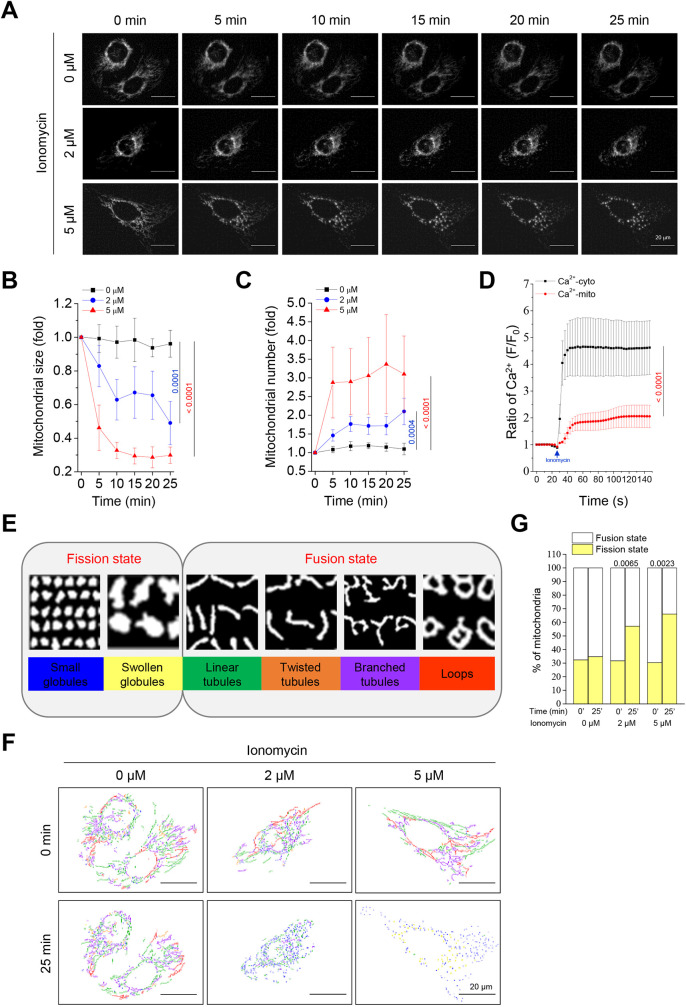
**Ionomycin induces the elevation in intracellular calcium (Ca^2+^) levels and mitochondrial fission.** Cytochrome *c* oxidase subunit 8A (COX8A)–mRFP overexpression in U2OS cells can be used to monitor dynamic changes in the morphology of mitochondria. (A) Real-time fluorescence imaging recorded the mitochondrial fission under ionomycin (0, 2 and 5 μM) treatment in 5-min intervals for 25 min. Images shown are representative of three biological repeats. Scale bars: 20 μm. (B,C) Quantitative analysis of mitochondrial average size (B) and number (C) from A, which were calculated using ImageJ at different time points and normalized against its initial state. (D) U2OS cells overexpressed plasmids expressing the cytoplasm-localized red Ca^2+^ indicator, R-GECO, and mitochondria-localized red Ca^2+^ indicator, LAR-GECO1.2-mt, to measure cytosolic (Ca^2+^-cyto) and mitochondrial Ca^2+^ (Ca^2+^-mito) levels, respectively. Cells were treated with 2 μM ionomycin at 30 s in Phenol Red-free DMEM. F/F_0_ indicates the mean fluorescence at time *t* divided by the mean fluorescence at time 0, and represents the change of relative Ca^2+^ concentration in the cell. (E) Classification of mitochondrial morphological states was defined using MicroP software. Mitochondria were allocated into six groups belonging to two states; different colors represent the groups of mitochondria. (F) Images from the initial point (0 min) and end point (25 min) under ionomycin (0, 2 and 5 μM) treatment were classified and the percentage of the different mitochondrial states is presented in Fig. S7A. Scale bars: 20 μm. (G) Statistical analysis of the mitochondrial morphological states using MicroP software. All values are represented as the mean±s.e.m. from three biological replicates with at least 15 cells per replicate, compared with the control group using one-way ANOVA with Dunnett's post hoc test; adjusted *P*-values are indicated for each comparison.

The role of intracellular Ca^2+^ in the regulation of mitochondrial morphology transitions has been examined in U2OS-COX8A–mRFP cells (cells with mRFP-labeled mitochondria). Time-lapse microscopy revealed that the addition of the Ca^2+^ ionophore ionomycin at concentrations of 2 and 5 μM resulted in the transition of mitochondrial morphology to round and globular structures (Fig. 1A). In experiments performed with 2–5 μM ionomycin, the morphological changes in mitochondria showed a significant decrease in size (Fig. 1B) and increase in number (Fig. 1C) compared with those of the control group (Movies 1 and 2). To observe the changes in Ca^2+^, the fluorescence protein-based Ca^2+^ indicators R-GECO and LAR-GECO1.2-mt were used as cytosolic and mitochondrial indicators, respectively. Addition of 2 μM ionomycin resulted in a 5-fold increase in cytosolic Ca^2+^ and a 2-fold increase in mitochondrial Ca^2+^ levels (Fig. 1D). Moreover, the concentration of cytoplasmic Ca^2+^ increased first, followed by an increase in Ca^2+^ in the mitochondria. We subsequently classified the end of the mitochondrial states using the analytic tool MicroP with the default conditions described previously (Peng et al., 2011), and mitochondria were classified into six groups based on their radius and length (Fig. 1E). Using MicroP, we assessed the mitochondrial state and classified the shape of the mitochondria in different colors (Fig. 1F; Fig. S7A). Our findings showed that a mitochondrial fission-like appearance occurred at the 25-min endpoint after stimulation with ionomycin (Fig. 1G). In contrast to ionomycin-induced Ca^2+^ elevation, BAPTA-AM strongly chelates intracellular Ca^2+^. Compared with the control group, BAPTA-AM treatment caused mitochondrial fragmentation (Movie 3; Fig. S1A) as well as a significant decrease in size (Fig. S1B) and increase in the number of mitochondria (Fig. S1C). Furthermore, BAPTA-AM caused the mitochondria to show fission-like morphology (Figs S1D,E and S7B). These data suggest that alterations in intracellular Ca^2+^ levels lead to a mitochondrial morphological transition.

### Phosphorylation of DRP1 at S616 increases Ca^2+^-induced mitochondrial fission

**Fig. 2.**
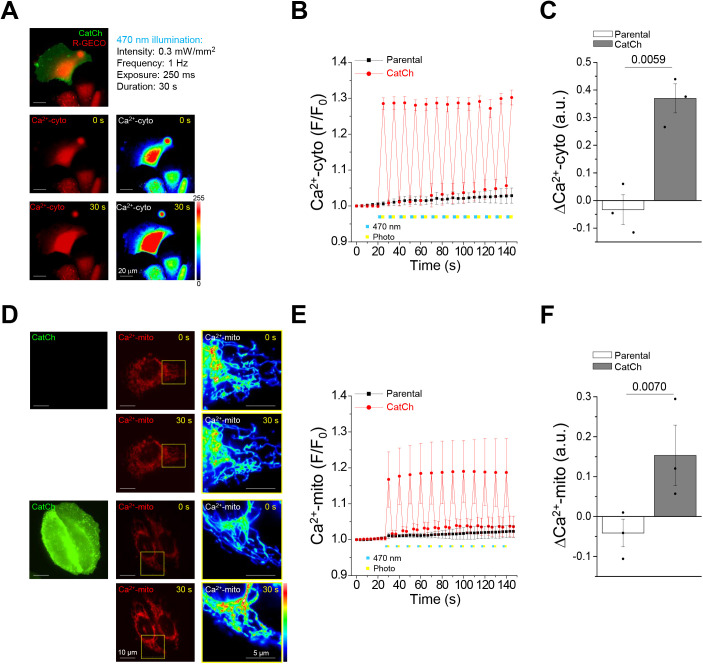
**Optogenetically engineered Ca^2+^ oscillation in the cytosol and mitochondria with time precision.** Cytosolic and mitochondrial Ca^2+^ oscillations can be triggered by optogenetics. (A,D) Fluorescence images of (A) Ca^2+^ translocating channelrhodopsin (CatCh)–Venus and R-GECO or (D) CatCh–Venus and LAR-GECO1.2-mt stable expression levels in U2OS cells. The fluorescence emission intensities of R-GECO and LAR-GECO1.2-mt indicating the cytosolic (Ca^2+^-cyto) and mitochondrial Ca^2+^ (Ca^2+^-mito) levels are represented by red fluorescence and enlarged pseudocolor images before (0 s) and after (30 s) 470 nm blue light illumination at 0.3 mW/mm^2^, 1 Hz and 250 ms exposure time for 30 s. Images shown are representative of three biological repeats. Scale bars: 20 μm (A); 10 μm (D); 5 μm (enlarged images in yellow squares in D). (B,E) The changes in (B) cytosolic Ca^2+^ and (E) mitochondrial Ca^2+^ levels in parental and CatCh-overexpressing U2OS cells were determined using a blue LED at a wavelength of 470 nm (blue squares), and red emission of R-GECO or LAR-GECO1.2-mt was excited using the green excitation light (yellow squares) with a wide-field microscope for Ca^2+^ recording. F/F_0_ indicates the mean fluorescence at time *t* divided by the mean at time 0, and represents the change of relative Ca^2+^ concentration in the cell. (C,F) Analysis of the maximum change in the increase in (C) cytosolic Ca^2+^ and (F) mitochondrial Ca^2+^ levels. All values are represented as the mean±s.e.m. from three biological replicates with at least 15 cells per replicate, compared with parental cells using one-way ANOVA with Dunnett's post hoc test; adjusted *P*-values are indicated for each comparison. a.u., arbitrary units.

**Fig. 3.**
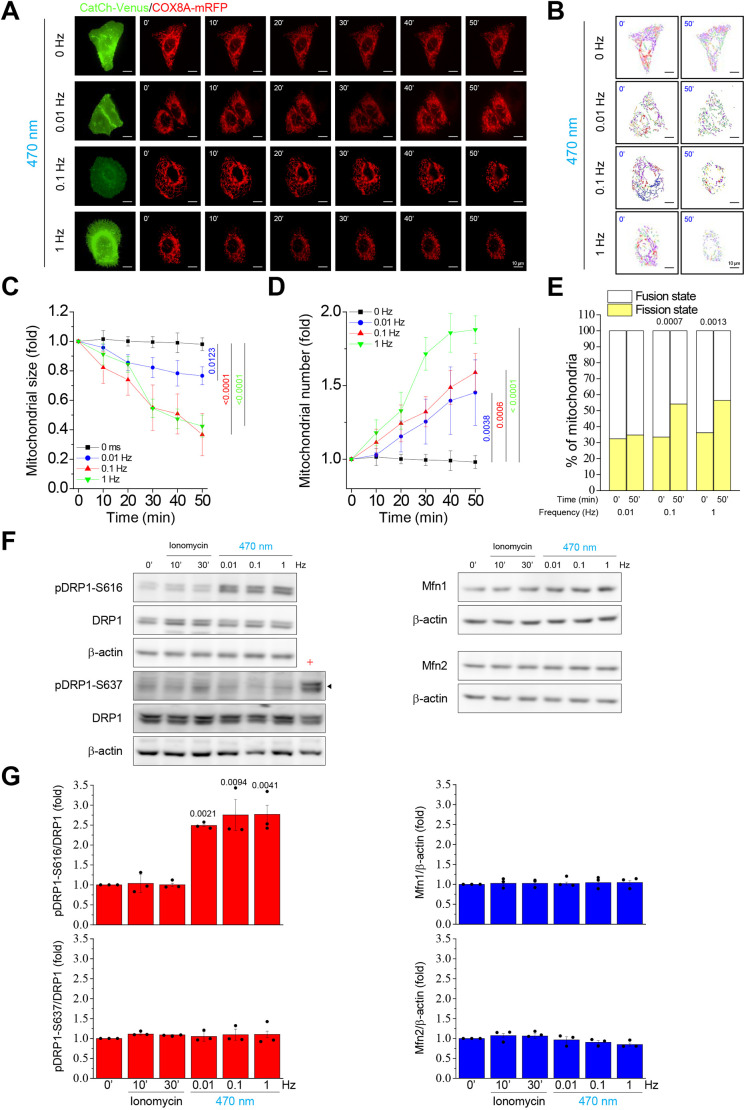
**Different frequencies of blue light illumination induce Ca^2+^ oscillations to modulate mitochondrial fission.** Real-time fluorescence images showing the dynamic changes in the mitochondrial network. The CatCh–Venus and COX8A–mRFP-overexpressing U2OS cells were subjected to 470 nm illumination (0.3 mW/mm^2^, 250 ms exposure time) at different frequencies (0, 0.01, 0.1 and 1 Hz) for 50 min. (A,B) Representative (A) fluorescence and (B) MicroP mitochondrial images. The percentages of the different mitochondrial states are presented in Fig. S7C. Images shown are representative of three biological repeats with at least 15 cells. Scale bars: 10 μm. (C,D) Quantitative analysis of mitochondrial average (C) size and (D) number from A were calculated using the ImageJ software at different time points and normalized against its initial state. (E) Images from the initial point (0 min) and end point (50 min) with light illumination at different frequencies were classified into fusion and fission states. Statistical analysis of mitochondrial states was performed using the MicroP software. All values are represented as the mean±s.e.m. from three biological replicates with at least 15 cells per replicate, compared with the control group using one-way ANOVA with Dunnett's post hoc test; adjusted *P*-values are indicated for each comparison. (F) Western blotting of phosphorylated dynamin-related protein 1 (DRP1) at serine 616 (pDRP1-S616) and serine 637 (pDRP1-S637), DRP1, mitofusin 1 (MFN1) and 2 (MFN2), and the internal control β-actin in whole-cell lysates. Forskolin-treated U2OS cells were used as the positive control for the phosphorylation of DRP1 at S637 (indicated by +). The immunoblots of whole membranes are presented in Fig. S8A. (G) Quantification of protein phosphorylation and expression as relative intensities of the blots are presented as the mean±s.e.m. from three biological replicates. *P*-values were calculated using two-tailed paired Student's *t*-test; adjusted *P*-values are indicated for each comparison.

In this study, modulation of intracellular Ca^2+^ oscillation influx was studied using CatCh-overexpressing U2OS-CatCh–Venus cells. To record Ca^2+^ oscillations, we applied R-GECO and LAR-GECO1.2-mt Ca^2+^ indicators co-transfected into U2OS-CatCh–Venus cells. Ca^2+^ imaging indicated that only cells expressing CatCh showed an increase in cytosolic (Fig. 2A) and mitochondrial (Fig. 2D) intensities under blue light illumination. Moreover, single-cell Ca^2+^ measurements showed that Ca^2+^ oscillation could be monitored based on changes in the fluorescence intensities of R-GECO (Fig. 2B) and LAR-GECO1.2-mt (Fig. 2E). Both cytosolic and mitochondrial Ca^2+^ levels increased by 0.4-fold (Fig. 2C) and 0.2-fold (Fig. 2F), respectively. More importantly, the data show that without illumination, the fluorescence intensities rapidly reverted to the initial levels, and they could be reactivated after further illumination. Thus, these data illustrate that the regulation of Ca^2+^ oscillation in cells can be precisely controlled by the optogenetic tool CatCh, depending on the illumination parameters (frequency, light intensity and exposure time). The mitochondrial morphology showed no significant changes at an illumination of 0.01 Hz within 50 min. Conversely, the groups that received 0.1 and 1 Hz illumination showed a mitochondrial morphological transition at the end of the illumination period (Fig. 3A). However, ImageJ assessments showed trends of mitochondrial fission with a significant decrease in size (Fig. 3C) and increase in number (Fig. 3D) in all illumination groups. Furthermore, MicroP analysis proved that the 0.1- and 1-Hz groups showed eventual fission states (Fig. 3B,E; Fig. S7C). To investigate the underlying molecular mechanism involved in the optogenetically engineered Ca^2+^-induced mitochondrial morphological transition, we focused on the protein levels of the mitochondrial fission protein DRP1 and mitochondrial fusion proteins MFN1 and MFN2. Western blotting results for the frequency-dependent assessments showed that the total levels of DRP1, MFN1 and MFN2 were the same as those in the control group (Fig. 3F,G). In addition, phosphorylation of DRP1 occurred only at S616 but not at S637. Interestingly, phosphorylation of DRP1 at S616 and S637 was not affected by ionomycin treatment. Subsequently, both light intensity (Figs S2 and S7D) and exposure time (Figs S3 and S7E) assessments yielded similar results to the frequency of light illumination shown in Fig. 3. Mitochondrial morphological transitions appeared with increased number and decreased size after illumination with light intensity at 0.1, 0.3 and 0.8 mW/mm^2^ (Fig. S2A,C,D) and exposure times of 100, 250 and 750 ms, respectively (Fig. S3A,C,D). In addition, our findings also showed that illumination induced mitochondrial to show fission-like morphology with light powers at 0.3 and 0.8 mW/mm^2^ (Figs S2B,E and S7D) and exposure times of 250 and 750 ms, respectively (Figs S3B,E and S7E). In contrast, the total levels of DRP1, MFN1 and MFN2 did not change under illumination with different power intensities and exposure times (Figs S2F,G and S3F,G). Phosphorylation only occurred at DRP1-S616 but not at DRP1-S637 after illumination with light power at 0.3 and 0.8 mW/mm^2^ (Fig. S2F,G) and exposure times of 250 and 750 ms, respectively (Fig. S3F,G). These data further validated that the optogenetically induced mitochondrial morphology transition is mediated by the phosphorylation of DRP1 at S616 instead of at S637 and occurs at higher frequencies, power intensities and exposure times.

### Ca^2+^-induced mitochondrial fission triggers DRP1 phosphorylation by the upstream Ca^2+^-dependent CaMKII, ERK1/2 and CDK1 pathways

**Fig. 4.**
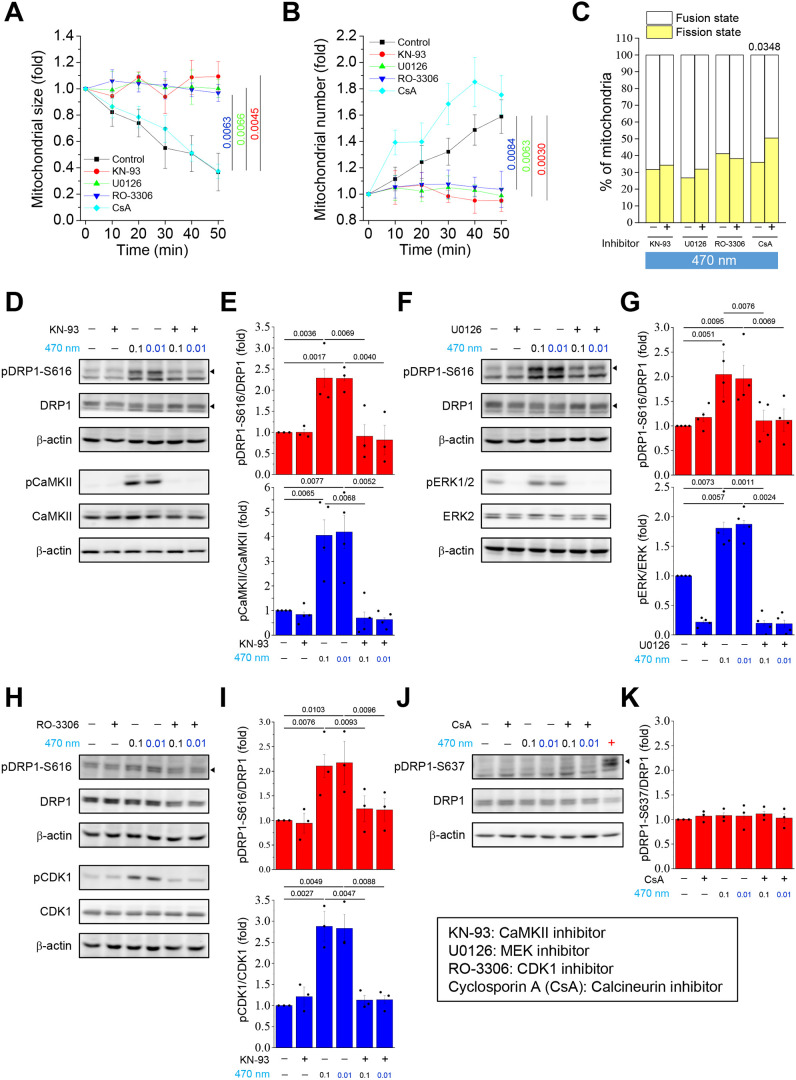
**Phosphorylation of DRP1 at S616 induces mitochondrial fission by optogenetically engineered Ca^2+^ oscillation.** The CatCh–Venus- and COX8A–mRFP-overexpressing U2OS cells under pretreatment with or without different inhibitors for 30 min, including 10 μM KN-93, 10 μM U0126, 10 μM RO-3306 and 10 μM cyclosporin A (CsA), followed by treatment with 470 nm illumination (0.3 mW/mm^2^, 0.1 Hz, 250 ms exposure time) for 50 min. (A,B) Quantitative analysis of mitochondrial average (A) size and (B) number using ImageJ at different time points. Each individual mitochondrion was normalized against its initial state. (C) Images from the initial point (0 min) and end point (50 min) with light illumination were classified into fusion and fission states. Statistical analysis of mitochondrial state was conducted using MicroP software. All values are represented as the mean±s.e.m. from three biological replicates with at least 15 cells per replicate, compared with the control group using one-way ANOVA with Dunnett's post hoc test; adjusted *P*-values are indicated for each comparison. (D,F,H,J) Western blotting of phosphorylated DRP1 at S616 (pDRP1-S616) and S637 (pDRP1-S637), phosphorylated Ca^2+^/calmodulin-dependent protein kinase II (pCaMKII), phosphorylated extracellular signal-regulated kinase 1/2 (pERK1/2), phosphorylated cyclin-dependent kinase 1 (CDK1) (pCDK1), DRP1, CaMKII, ERK2, CDK1, and the internal control β-actin in whole-cell lysates. The immunoblots of whole membranes are presented in Fig. S8B. In J, forskolin-treated U2OS cells were used as the positive control for phosphorylation of DRP1 at S637. (E,G,I,K) Quantification of protein phosphorylation and expression. Relative intensities of the blots are presented as the mean±s.e.m. from three biological replicates. *P*-values were calculated using two-tailed paired Student's *t-*test; adjusted *P*-values are indicated for each comparison.

We specifically focused on the Ca^2+^-related signaling pathways involved in DRP1 phosphorylation. Among the selected pathways, the CaMKII, ERK1/2 and CDK1 pathways were specialized upstream signal pathways for phosphorylation of DRP1-S616, whereas the Ca^2+^-mediated calcineurin pathway was upstream for dephosphorylation of DRP1-S637. Although phosphorylation of DRP1-S637 tended to inhibit mitochondrial fission and DRP1-S637 was shown to have no relationship with optogenetically induced mitochondrial fission (Fig. 3; Figs S2 and S3) (Ko et al., 2016), time-lapse images and MicroP analysis clearly showed that under the same illumination conditions, mitochondrial fission was avoided in groups pretreated with KN-93, U0126 and RO-3306, which are inhibitors of CaMKII, MEK1/2 and CDK1, respectively (Fig. S4A–C). In contrast, mitochondrial fission occurred after illumination in groups pretreated with CsA, a calcineurin inhibitor (Fig. S4D). ImageJ assessments showed that the trends of light-induced Ca^2+^-mediated mitochondrial fission with a significant decrease in size (Fig. 4A) and increase in number (Fig. 4B) were inhibited in the KN-93, U0126 and RO-3306 groups, and MicroP analysis proved that the above groups showed no changes in their fusion and/or fission states (Fig. 4C). In contrast, pre-treatment with CsA did not prevent Ca^2+^-mediated mitochondrial fission. Western blotting results for the frequency-dependent assessments showed that the total levels of DRP1, MFN1 and MFN2 were the same as those in the control non-illumination groups (Fig. 4D–K). Remarkably, light illumination-mediated Ca^2+^ oscillations increased the activation and phosphorylation of CaMKII, ERK1/2 and CDK1 (Fig. 4D–I). Furthermore, pretreatment with KN-93, U0126 and RO3306 completely inhibited the phosphorylation of CaMKII, ERK1/2 and CDK1, respectively. In addition, phosphorylation of DRP1 occurred only at S616 but not at S637 under blue light illumination. Unlike phosphorylation at S616, phosphorylation at S637 of DRP1 inhibited mitochondrial fission. Calcineurin was activated by elevated cytosolic Ca^2+^ levels. Activated calcineurin can dephosphorylate DRP1-S637, and dephosphorylated DRP1 further facilitates mitochondrial fission (Yu et al., 2019). The results of the present study showed that DRP1-S637 was not phosphorylated in optogenetics-induced mitochondrial fission (Fig. 4J,K). NFAT is a downstream target of calcineurin and can be dephosphorylated by activated calcineurin. To verify the effect of CsA on calcineurin inhibition, we examined the ability of CsA to prevent thapsigargin-induced NFAT dephosphorylation (Fig. S4E), which showed that CsA can effectively inhibit the phosphatase activity of calcineurin, thereby inhibiting the dephosphorylation of NFAT by calcineurin. Overall, Ca^2+^-induced mitochondrial fission by optogenetics occurs through phosphorylation of DRP1-S616, controlled by upstream ERK, CaMKII and CDK1 pathways, instead of through dephosphorylation of DRP1-S637.

### Optogenetics-induced mitochondrial fission promotes phosphorylated DRP1-S616 colocalization in mitochondria

**Fig. 5.**
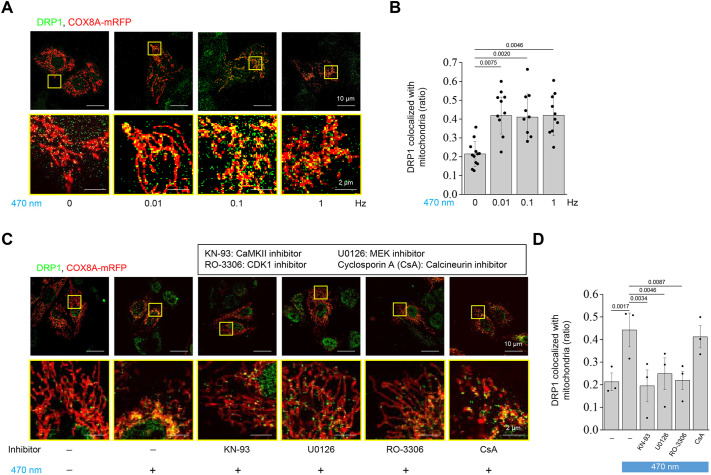
**Translocation of DRP1 to mitochondria is involved in light illumination-induced mitochondrial fission.** The CatCh–Venus- and COX8A–mRFP-overexpressing U2OS cells were treated with 470 nm illumination (0.3 mW/mm^2^, 250 ms exposure time) at different frequencies (0, 0.01, 0.1 and 1 Hz) for 50 min. Immunostaining was performed to label DRP1, and the fluorescence images were obtained using confocal microscopy. Enlarged images are shown in yellow squares. COX8A–mRFP was used as the mitochondrial marker. Scale bars: 10 μm (upper panels); 2 μm (lower panels). (A) Representative fluorescence images of DRP1 (green) and mitochondria (red) from three biological repeats. (B) Quantitative analysis of DRP1 colocalized with mitochondria under different frequencies of light illumination was performed using the JACoP plugin in ImageJ software. (C) The CatCh–Venus- and COX8A–mRFP-overexpressing U2OS cells under pretreatment with or without different inhibitors, including 10 μM KN-93, 10 μM U0126, 10 μM RO-3306 and 10 μM CsA, for 30 min, followed by treatment with 470 nm illumination (0.3 mW/mm^2^, 0.1 Hz, 250 ms exposure time) for 50 min. Representative fluorescence images of DRP1 (green) and mitochondria (red) from three biological repeats. (D) Quantitative analysis of DRP1 colocalized with mitochondria under different inhibitors using the JACoP plugin in ImageJ software. All values are represented as the mean±s.e.m. from three biological replicates with at least 15 cells per replicate using two-tailed paired Student's *t*-test; adjusted *P*-values are indicated for each comparison.

**Fig. 6.**
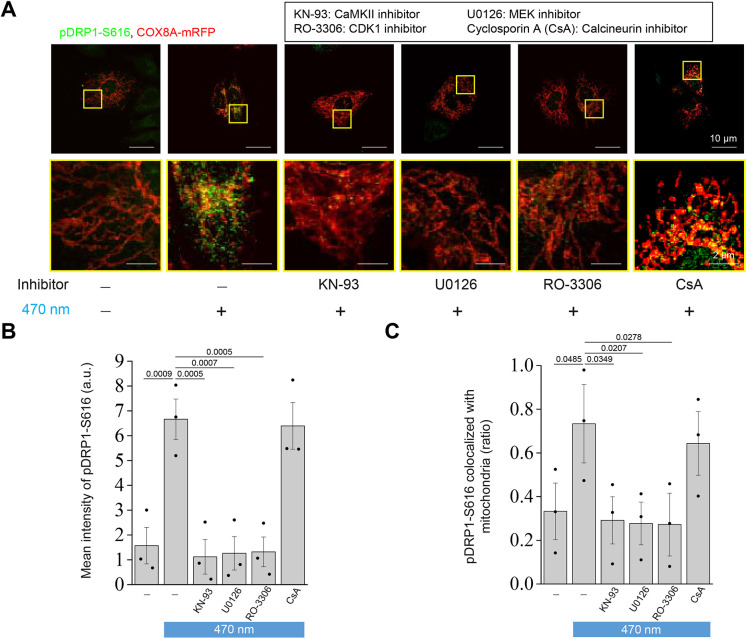
**Translocation of pDRP1 at S616 to mitochondria is involved in light illumination-induced mitochondrial fission.** CatCh–Venus- and COX8A–mRFP-overexpressing U2OS cells under pretreatment with or without different inhibitors, including 10 μM KN-93, 10 μM U0126, 10 μM RO-3306 and 10 μM CsA, for 30 min, followed by treatment with 470 nm illumination (0.3 mW/mm^2^, 0.1 Hz, 250 ms exposure time) for 50 min. Immunofluorescence staining was performed to label pDRP1 at S616 (pDRP1-S616), and fluorescence images were obtained using confocal microscopy. COX8A–mRFP was used as the mitochondrial marker. (A) Representative fluorescence images of pDRP1-S616 (green) and mitochondria (red) from three biological replicates. Enlarged images are shown in yellow squares. Scale bars: 10 μm (upper panels); 2 μm (lower panels). (B,C) Quantitative analysis of the (B) mean fluorescence intensity of pDRP1-S616 and (C) proportion of pDRP1-S616 colocalized with mitochondria under different treatments was performed using the JACoP plugin in ImageJ software. All values are represented as the mean±s.e.m. from three biological replicates with at least 15 cells per replicate using one-way ANOVA with Dunnett's post hoc test; adjusted *P*-values are indicated for each comparison.

During mitochondrial fission, DRP1 is recruited to the mitochondria to slice tubule-like mitochondria into globular bodies. We examined the colocalization between mitochondria and DRP1, and found that illumination at 0.01, 0.1 and 1 Hz resulted in greater recruitment of DRP1 to mitochondria in comparison with that in the control group (Fig. 5A). The colocalization analysis tool JaCoP from ImageJ suggested recruitment of DRP1 to mitochondria in the illumination groups with a Mander's overlap coefficient (MOC) of approximately 0.4; in contrast, the control group had an MOC of approximately 0.2 (Fig. 5B). Interestingly, the inhibitors of CaMKII, ERK1/2 and CDK1, but not calcineurin, also inhibited DRP1 colocalization with mitochondria (Fig. 5C,D). Furthermore, we stained the phosphorylated mitochondrial fission protein DRP1 at S616 (pDRP1-S616) to observe the mean intensity and localization of pDRP1-S616. Following the application of KN93, U0123 or RO-3306 with illumination, confocal images showed a reduction in the fluorescence intensity of pDRP1-S616 compared with that in the illumination group (Fig. 6A,B). However, CsA did not inhibit the optogenetically induced DRP1 phosphorylation at S616. Moreover, pDRP1-S616 was recruited to the mitochondria only under illumination with or without CsA pretreatment (Fig. 6C). These data illustrate that after illumination, DRP1 is phosphorylated at S616 and recruited to the mitochondria, which eventually facilitates mitochondrial fission.

### Optogenetics-induced mitochondrial dysfunction, autophagy and apoptosis

**Fig. 7.**
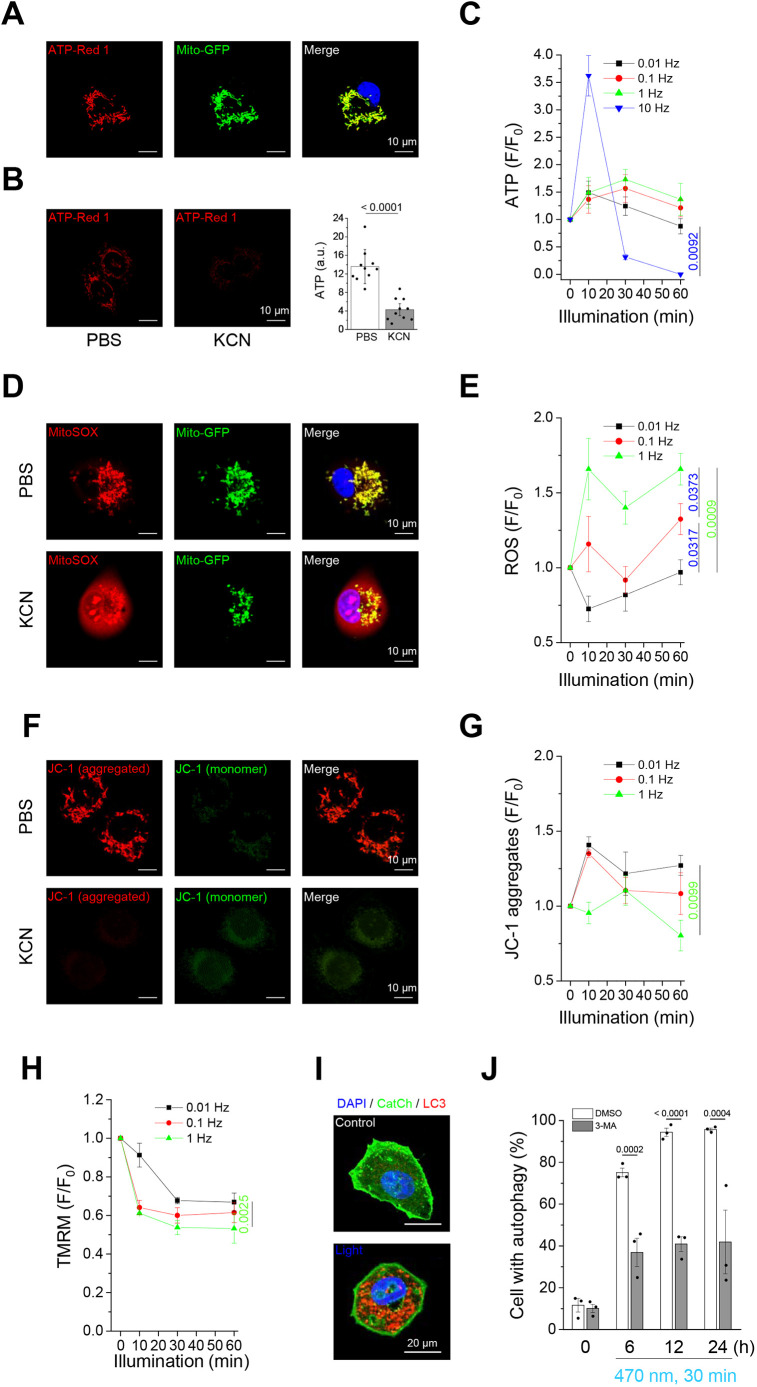
**High-frequency Ca^2+^ oscillations induce mitochondrial dysfunction.** Real-time fluorescence images showed dynamic changes in mitochondrial ATP, superoxide levels and membrane potential. CatCh–Venus-overexpressing U2OS cells were stained with a mitochondrial ATP indicator (5 μM ATP-Red 1), superoxide indicator (5 μM MitoSOX Red) or membrane potential indicators (5 μg/ml JC-1 and 50 nM TMRM) under treatment with 470 nm illumination (0.3 mW/mm^2^, 250 ms exposure time) at different frequencies (0, 0.01, 0.1 and 1 Hz) for 60 min. (A,B,D,F) Representative confocal images of (A,B) ATP-Red 1, (D) MitoSOX, (F) JC-1 and (A,D) fluorescent protein-tagged mitochondria (Mito–GFP) when cells were treated with or without KCN for 30 min. Mito-GFP was used as the mitochondrial marker, whereas KCN was used for mitochondrial ATP depletion. Scale bars: 10 μm. (B,C,E,G,H) Quantitative analysis of mitochondrial (B,C) ATP, (E) superoxide levels and (G,H) membrane potential under 470 nm illumination at different time points using flow cytometry. All values are represented as the mean±s.e.m. from three biological replicates using one-way ANOVA with Dunnett's post hoc test; adjusted *P*-values are indicated for each comparison. F/F_0_ indicates the mean fluorescence at time *t* divided by the mean fluorescence at time 0. (I) CatCh–Venus-overexpressing U2OS cells were imaged following 6, 12 or 24 h of incubation after 470 nm illumination (0.3 mW/mm^2^, 0.1 Hz, 250 ms exposure time) for 30 min. Immunofluorescence staining was performed to label LC3, and the fluorescence images were obtained using confocal microscopy. DAPI was used as the nucleus marker. Representative images at the 6 h timepoint are shown. Scale bars: 20 μm. (J) Quantitative analysis of the autophagic cells after 470 nm illumination with or without autophagy inhibitor (5 mM 3-MA) pretreatment. All values are represented as the mean±s.e.m. from three biological replicates using two-tailed paired Student's *t*-test; adjusted *P*-values are indicated for each comparison.

**Fig. 8.**
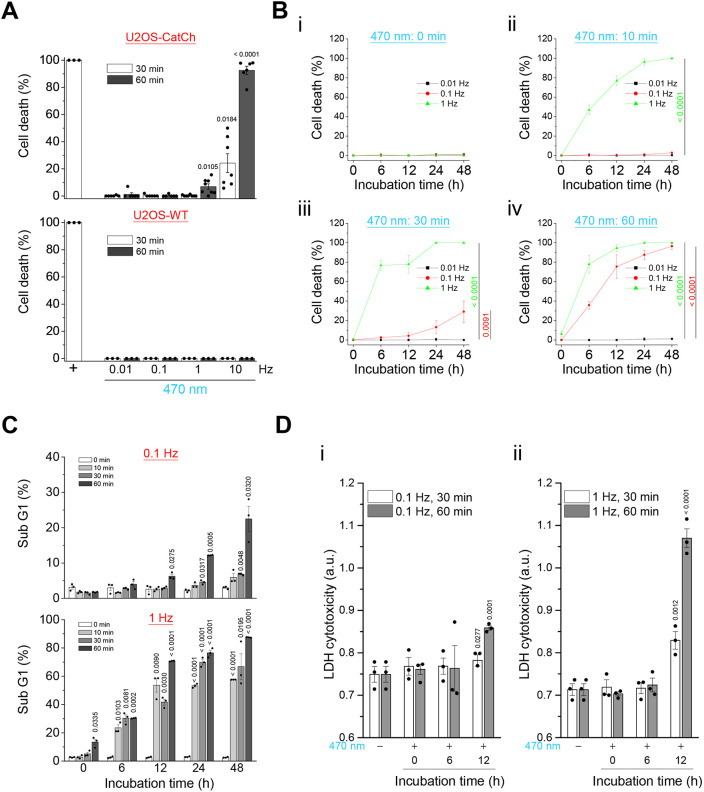
**High-frequency Ca^2+^ oscillations induce cytotoxicity and cell death.** (A,B) Live- and dead-cell analysis of the parental U2OS cells (U2OS-WT) and CatCh-overexpressing U2OS cells (U2OS-CatCh) after 470 nm illumination. (A) Quantitative analysis of the percentage of cell death immediately after 30 or 60 min of 470 nm illumination at different frequencies (0.01, 0.1, 1 and 10 Hz), fixed 0.1 mW/mm^2^ power and 100 ms exposure time. All values are represented as the mean±s.e.m. from three biological replicates using two-tailed paired Student's *t*-test; adjusted *P*-values are indicated for each comparison. (B) Quantitative analysis of the percentage of cell death at different time points (0, 6, 12, 24 and 48 h) after (i) 0, (ii) 10, (iii) 30 or (iv) 60 min of 470 nm illumination at different frequencies (0.01, 0.1 and 1 Hz), fixed 0.1 mW/mm^2^ power, and 100 ms exposure time. All values are represented as the mean±s.e.m. from three biological replicates using one-way ANOVA with Dunnett's post hoc test; adjusted *P*-values are indicated for each comparison. (C) Propidium iodide (PI) staining of fixed cells for flow cytometry analysis was used to evaluate the apoptotic cells in sub-G1 phase. The percentage of cells in sub-G1 phase was analyzed at different time points (0, 6, 12, 24 and 48 h) after 0, 10, 30 or 60 min of 470 nm illumination at 0.1 and 1 Hz, fixed 0.1 mW/mm^2^ power and 100 ms exposure time. (D) LDH cytotoxicity analysis of U2OS-CatCh cells at different time points (0, 6 and 12 h) after 470 nm illumination for 30 or 60 min at (i) 0.1 Hz or (ii) 1 Hz, fixed 0.1 mW/mm^2^ power and 100 ms exposure time. All values are represented as the mean±s.e.m. from three biological replicates using two-tailed paired Student's *t*-test; adjusted *P*-values are indicated for each comparison.

Mitochondria are essential organelles that regulate cellular energy homeostasis, oxidative stress and cell death. The above results showed that optogenetically engineered Ca^2+^ oscillations induced mitochondrial fission, which might lead to mitochondrial dysfunction and cell death. ATP-Red 1, a mitochondrial ATP fluorescence probe, was shown to colocalize with the mitochondria by confocal microscopy (Fig. 7A). We observed the inhibition of mitochondrial electron transfer chain complexes by potassium cyanide (KCN)-induced mitochondrial ATP depletion (Fig. 7B). The higher the Ca^2+^ concentration in the mitochondria, the better the synthesis of ATP within the normal range of Ca^2+^ concentration. Our results showed that higher-frequency illumination resulted in relatively high ATP synthesis. However, although mitochondria could synthesize a large amount of ATP in a short duration of 10 min, the subsequent ATP synthesis could not be achieved if the illumination frequency was too high (10 Hz) (Fig. 7C). Excessive ROS production and loss of membrane potential in mitochondria are important indicators of mitochondrial damage and loss of function. ATP production in the mitochondrial electron transfer chain is accompanied by ROS production. Using MitoSOX Red to display the ROS produced by mitochondria, we found that the higher the frequency of illumination, the more ROS that were produced (Fig. 7D,E). In contrast, both confocal imaging and flow cytometry analysis showed that the higher the frequency of illumination, the lower the mitochondrial membrane potential by tetraethylbenzimidazolylcarbocyanine iodide (JC-1) and tetramethylrhodamine methyl ester (TMRM) staining (Fig. 7F–H). KCN was used as a positive control to induce ROS formation and decrease mitochondrial membrane potential. Microtubule-associated protein light chain 3 (LC3) is widely used as an autophagy marker in assays that measure autophagic activity. To verify this phenomenon, we used anti-LC3 immunofluorescence staining to observe autophagic activity. Confocal microscopy images showed that light-induced Ca^2+^ oscillation significantly increased the puncta of LC3-positive cells (Fig. 7I). Induction of autophagy by illumination was significantly increased; 75% of cells were observed to form LC3 puncta after 30 min of light and then incubation for 6 h, reaching 95% after 12 h (Fig. 7J). In contrast, pretreatment of the cells with 3-MA, an autophagy inhibitor, significantly reduced light-induced autophagy (Fig. 7J). To further examine Ca^2+^-induced cell death, we used live- and dead-cell staining, hypoploidy analysis, and lactate dehydrogenase (LDH) cytotoxicity assay for cell death evaluation. Calcein-AM and ethidium-1 were used to distinguish live and dead cells, respectively. As a positive control for dead cells, U2OS wild-type (WT) cells were fixed with 4% paraformaldehyde and became dead cells (Fig. S5A). For Ca^2+^-induced cell death analysis using optogenetics, we stimulated the U2OS-CatCh–Venus cell line with four different frequencies (0.01, 0.1, 1 and 10 Hz) of blue light for 30 and 60 min to investigate the effect of frequency on cell death. We demonstrated that a higher frequency of Ca^2+^ oscillations induced cell death after light illumination, whereas longer illumination times also caused more obvious cell death (Fig. S5B,C). U2OS-WT cells were used to demonstrate that Ca^2+^ oscillations are the main factor in cell death by optogenetics. The results showed that no cells died after illumination, even when stimulated at 10 Hz (Fig. S6). This showed that light-stimulated cell death in U2OS-CatCh–Venus was caused by Ca^2+^ oscillations but not by heat and phototoxicity produced during light illumination. First, we assessed cell death immediately after light illumination. More than 10% of the cells died after 10 Hz stimulation after 30 min of light illumination, whereas 0.01, 0.1 and 1 Hz stimulations did not induce cell death. After 60 min of light illumination, more than 70% of the cells died after stimulation at 10 Hz. In addition, 1 Hz induced more than 5% cell death after light stimulation (Fig. 8A). For long-term cultures (6, 12, 24 and 48 h) of cells after illumination for different durations (10, 30 and 60 min), the rate of cell death increased in a frequency- and duration-dependent manner (Fig. 8B). Propidium iodide (PI) staining of DNA for sub-G1 hypoploidy analysis can be used to quantify apoptosis. Based on the results from similar experimental designs shown in Fig. 8B, the percentage of the sub-G1 phase increased in a time-dependent manner (Fig. 8C). There was no significant increase in the sub-G1 phase during light illumination (0.1 Hz), whereas only 20% of the sub-G1 phase was observed after 48 h of incubation under light illumination for 60 min. In contrast, the sub-G1 phase significantly increased gradually in an incubation time-dependent manner after illumination at 1 Hz (Fig. 8C). Finally, an LDH cytotoxicity assay was used to examine Ca^2+^ cytotoxicity caused by optogenetics. Cytotoxicity was significantly increased after 12 h of incubation with light illumination at 1 Hz for 30 or 60 min, which indicates the leakage of LDH out of the cells caused by cell necrosis (Fig. 8D). In contrast, only minor increases were observed after 12 h of incubation with light illumination at 0.1 Hz.

## DISCUSSION

Optogenetic stimulation differs from traditional methods, such as chemical, electrical and physical stimulation, and its greatest advantage lies in its precise control over spatial and temporal resolutions (Deisseroth, 2011). The optogenetic system allows for minute and precise control of cytosolic Ca^2+^ oscillations. In this study, we chose various optical parameters (frequency, intensity and exposure time) to mimic and achieve the specified Ca^2+^ oscillations, thereby inducing target signal pathways. Our study proved that the optogenetic molecular tool CatCh can trigger Ca^2+^ influx, and then enter the mitochondria and cause physiological or pathological changes (Fig. S2). Excessive Ca^2+^ can cause mitochondrial fission (Fig. 3; Figs S2 and S3), excessive ROS production, decreased membrane potential, autophagy (Fig. 7) and cell death (Fig. 8).

Ca^2+^ concentration can regulate critical physiological events in cells. In this study, we mimicked Ca^2+^ influx by applying ionomycin and chelating Ca^2+^ with BAPTA-AM. A previous study showed that mitochondrial dynamics are intimately linked to changes in Ca^2+^ concentration changes (Youle and van der Bliek, 2012). Mitochondrial fission occurred rapidly to engage with environmental changes in Ca^2+^ levels in both ionomycin and BAPTA-AM challenges (Fig. 1; Fig. S1). Our findings show that the parameters used in the optogenetic platform can be controlled to promote mitochondrial fission. For example, all frequencies (0.01, 0.1 and 1 Hz) of illumination caused mitochondrial fission, but the effect caused by 0.01 Hz was relatively weak (Fig. 3). With respect to light intensity, a high light intensity of 0.8 mW/mm^2^ activated mitochondrial fission faster than 0.3 mW/mm^2^, but fission was not seen at 0.1 mW/mm^2^ (Fig. S2). Finally, in assessments based on the exposure time, a 100-ms illumination period appeared to have a minor effect on mitochondrial fission; however, mitochondrial fission was easily observable over an exposure time of 250 and 750 ms (Fig. S3). These observations proved that with the optogenetic method, the use of higher light intensity, frequency and exposure time of illumination might lead to the accumulation of higher Ca^2+^ in cells, which, in turn, causes mitochondrial fission.

We attempted to identify the unique molecular mechanism that activates mitochondrial fission by Ca^2+^ signaling, for which we selected Ca^2+^-activated upstream candidates of DRP1. ERK, CaMKII and CDK1 were reported to show a close relationship with DRP1-S616 phosphorylation and subsequent induction of mitochondrial fission (Roe and Qi, 2018). In contrast, calcineurin was reported to induce dephosphorylation at DRP1-S637 and was also well explored in our study (Cribbs and Strack, 2007). Our results revealed that the optogenetic method could activate phosphorylation of ERK, CaMKII and CDK1, and DRP1 at S616. In contrast, the dephosphorylation of DRP1-S637 was not readily observable with illumination or even after pretreatment with CsA. These findings suggest that optogenetically induced mitochondrial fission does not involve DRP1-S637, but is instead mediated by ERK, CaMKII and CDK1 and their downstream phosphorylation target DRP1-S616 (Fig. 4). In addition, optogenetically engineered Ca^2+^ signaling leads to mitochondrial localization of phosphorylated DRP1-S616, which appears to be a key step in mitochondrial fission (Figs 5 and 6).

Ionomycin is an effective Ca^2+^ ionophore that is commonly used to modify intracellular Ca^2+^ concentrations. It can increase and sustain Ca^2+^ concentration, called ‘steady-state Ca^2+^’ (leaving the initial peak Ca^2+^ response unaffected), but not Ca^2+^ oscillation. In this study, we found that illumination triggered Ca^2+^ oscillation by phosphorylation at S616 but not dephosphorylation at S637 of DRP1 via the activation of the Ca^2+^-dependent kinases CaMKII, ERK and CDK1 (Figs 3F,G and 4D–I). However, ionomycin had no effect on the phosphorylation of DRP1 at S616 and dephosphorylation at S637 (Fig. 3F,G). Therefore, we propose that optogenetic engineering plays a critical role in Ca^2+^ oscillation but not on steady-state Ca^2+^ levels by ionomycin.

By comparing live- and dead-cell staining, hypoploidy analysis and LDH cytotoxicity results in short- and long-term incubations after light illumination, we speculated that Ca^2+^ caused cell death through necrotic and apoptotic pathways. Illumination at 10 Hz for 30 min immediately caused failure in ATP production and necrosis of some cells (Figs 7C and 8A), whereas more than 80% of cells underwent necrosis when illuminated for 60 min (Fig. 8A). In contrast, 1 Hz illumination within 60 min resulted in a marked increase in mitochondrial ROS, a decrease in mitochondrial membrane potential and a small amount of cell death (Figs 7D–H and 8A). Furthermore, extensive cell death (Fig. 8B) and increased LDH cytotoxicity (Fig. 8D) were observed after 6 h of incubation, and an increased proportion of cells in the sub-G1 phase was also observed (Fig. 8B) after illumination at 1 Hz for 10, 30 or 60 min. Thus, we can infer that cell death caused by 1 Hz illumination is predominantly through apoptosis. In contrast, 0.1 Hz illumination only caused autophagy and a very small proportion of cells to die by apoptosis after long-term illumination and incubation (Figs 7J and 8), whereas 0.01 Hz illumination did not cause damage to the cells (Fig. 8). Different cell types respond to Ca^2+^ waves at different frequencies (Yu et al., 2019). In previous studies, it was found that the frequency of Ca^2+^ oscillation under physiological conditions mostly ranges from 0.01 to 0.1 Hz, and the frequency range of Ca^2+^ oscillation that causes cell damage or death is 1 to 10 Hz (Smedler and Uhlén, 2014; Wacquier et al., 2019); so, the results of this study are consistent with previous related studies.

Mdivi-1, an inhibitor of DRP1 translocation, inhibits DRP1, leading to reduced mPTP-induced neurotoxicity in mice (Rappold et al., 2014). Recent studies have shown that DRP1 is recruited next to mPTP under hypoxia, resulting in mitochondrial fragmentation and structural damage owing to excessive opening of mPTP, leading to cell death. Interestingly, they found that CsA prevented mPTP opening without reducing DRP1 mitochondrial translocation, which might not interfere with the process of mitochondrial fission during hypoxia (Duan et al., 2021). Furthermore, a study found that chronic administration of isoproterenol persistently enhanced the frequency of mPTP openings, followed by mitochondrial damage and cardiac dysfunction, which were mediated by the phosphorylation of DRP1-S616 by Ca^2+^/CaMKII (Xu et al., 2016). These studies demonstrate that even CsA plays an important role in mPTP opening. However, DRP1 acts upstream of mPTP, which is an essential factor for mitochondrial fission. In this study, we investigated the effect of optogenetics on Ca^2+^ oscillation. Therefore, we assumed that the calcineurin signaling pathway might be involved in DRP1-mediated mitochondrial fission. Thus, we pre-treated the cells with CsA as a calcineurin inhibitor under light stimulation. However, we did not observe CsA blocking Ca^2+^-mediated mitochondrial fission or dephosphorylation of DRP1-S637. Interestingly, mitochondrial fission induced by optogenetic treatment is related to DRP1-S616 via ERK, CaMKII and CDK1, but has no impact on DRP1-S637.

Different intracellular Ca^2+^ oscillatory waves affect multiple cellular functions and can be combined from specific modes of frequency, amplitude, duty cycle, duration and location. Previous studies have found that different cells and molecules respond differently to different Ca^2+^ oscillations (Colella et al., 2008; Smedler and Uhlén, 2014; Hannanta-Anan and Chow, 2016) through a process called frequency or amplitude decoding. Molecules that can be regulated by Ca^2+^ and have different degrees of response to Ca^2+^ depend on the number of domains that can bind to Ca^2+^ and their affinity for Ca^2+^. The transcriptional activity of Ca^2+^-dependent transcription factors is regulated by Ca^2+^ oscillations (Dolmetsch et al., 1997; Boulware and Marchant, 2008; Parekh, 2011; Song et al., 2012; Smedler and Uhlén, 2014). Our previous study found that NFAT was significantly activated under illumination with high-frequency or -power intensity light. In contrast, NFκB showed significant activation under illumination with high-frequency or -power intensity light (Lai et al., 2021). In this study, we found that the phosphorylation of DRP1 at S161 can be activated by higher frequency, stronger power intensity and longer exposure time to illumination conditions (Fig. 3, Figs S2 and S3), which might lead to higher Ca^2+^ concentrations within the cells. However, the continuous influx of intracellular Ca^2+^ induced by ionomycin failed to induce phosphorylation of DRP1 at S616, which might have been caused by the high concentration of Ca^2+^ or lack of Ca^2+^ oscillations (Fig. 3, Figs S2 and S3).

This study provides a novel method for the regulation of mitochondrial dynamics. By precisely controlling the Ca^2+^ oscillations using optogenetics, we can manipulate the mitochondrial dynamics. This research platform can be used for the in-depth study of the molecular mechanisms associated with mitochondrial fission, and to screen for related molecules with protective effects to avoid mitochondrial fission.

## MATERIALS AND METHODS

### Cell culture and chemical reagents

The human bone osteosarcoma cell line U2OS was maintained in low-glucose Dulbecco's modified Eagle's medium (DMEM; Gibco, 12491015; MT, USA) supplemented with 10% fetal bovine serum (FBS; Gibco, 10437028, MT, USA), penicillin-streptomycin (100 μg/ml; Simply Biologics, CC502-0100, NV, USA) in 5% CO_2_ at 37°C. U0126, cyclosporin A, ATP-Red 1, thapsigargin, ionomycin, BAPTA-AM and KCN were purchased from Sigma-Aldrich (St. Louis, MO, USA). MitoSOX Red, TMRM and 4′,6-diamidino-2-phenylindole dihydrochloride (DAPI) were purchased from Invitrogen (Carlsbad, CA, USA). KN-93 and 3-MA were purchased from Cayman (Michigan, MI, USA). JC-1 and RO-3306 were purchased from BioVision (Milpitas, CA, USA) and Santa Cruz Biotechnology (Santa Cruz, CA, USA), respectively. Information on chemical reagents can be found in Table S1.

### DNA transfection

U2OS cells were transfected with Venus-tagged CatCh (CatCh–Venus), mRFP-tagged cytochrome *c* oxidase subunit 8A (COX8A) (COX8A–mRFP), GFP-tagged porin (Mito–GFP), LAR-GECO1.2-mt and R-GECO plasmids using Lipofectamine 3000 (Invitrogen, L3000015, San Diego, CA, USA), and used for experiments 48 h later. Stable clones were selected using 500 µg/ml geneticin (G418; Gibco, 11811098, Big Cabin, OK, USA) and sorted using flow cytometry (FACSAria; BD Biosciences, San Jose, CA, USA). To maintain the expression levels of these fluorescent protein-tagged proteins, cells were sorted once every 2 months. Information on plasmids can be found in Table S1.

### Optogenetic platform

An LED illumination system (Thorlabs, NJ, USA) supplied 470 nm blue light, and the DC2100 software connected to a function generator was used to manipulate the optical parameters (light intensity, frequency, exposure time and duration). According to the experimental requirements, a single 470 nm LED light source could be operated under a microscope to detect fluorescence changes in real time after blue light illumination of the cells. A customized 470 nm LED array light box consisting of 42 high-power (1 W) LED bulbs was used to irradiate large areas of cell samples for protein collection, and a power meter device (OPHIR NOVA II, Israel) was used to measure the LED light output power.

### Single-cell intracellular Ca^2+^ measurements

Changes in the fluorescence intensity related to intracellular Ca^2+^ levels in living U2OS cells were measured using a single-cell fluorimeter (Till Photonics, Germany). The fluorescent protein-based Ca^2+^ indicators R-GECO (cytosolic Ca^2+^ indicator) and LAR-GECO1.2-mt (mitochondrial Ca^2+^ indicator) were used as intracellular Ca^2+^ probes, with excitation at a wavelength of 560 nm. R-GECO and LAR-GECO1.2-mt were transfected into CatCh–Venus-overexpressing U2OS cells prior to blue light illumination. The fluorescence emission intensity was monitored at 590 nm, stored digitally and analyzed using the software TILLvisION 4.0 (Till Photonics, Germany).

### Western blotting

Cell lysates were harvested in radioimmune precipitation assay (RIPA) buffer containing 150 mM NaCl, 10 mM EDTA, 50 mM Tris-HCl at pH 7.4, 1% NP-40, 0.004% sodium azide, 0.5% sodium deoxycholate, 0.1% SDS and protease inhibitor cocktail (Roche cOmplete, 04693132001, Switzerland) supplemented with 1 mM NaF, 1 mM PMSF and 1 mM Na_3_VO_4_. Whole-cell-lysate proteins were separated by SDS-PAGE and electroblotted onto nitrocellulose membranes, which were incubated with several primary antibodies, including anti-nuclear factor of activated T cells (NFAT) [diluted 1:1000 in TBS-T buffer (0.1 M Tris, 1 M NaCl and 0.05% Tween-20, pH 7.6); Invitrogen, MA3-024], anti-LC3 (1:1000; Invitrogen, PA1-16931), anti-phospho-ERK1/2 (1:1000; arigo Biolaboratories, ARG52277, Taiwan), anti-ERK2 (1:1000; arigo Biolaboratories, ARG62350), anti-CaMKII (1:1000; ABclonal #A2508, MA, USA), anti-phospho-DRP1-S616 (1:1000; Cell Signaling Technology, 3455, MA, USA), anti-phospho-DRP1-S637 (1:1000; Cell Signaling Technology, 6319), anti-phospho-CaMKII (1:1000; Cell Signaling Technology, 12716), anti-MFN1 (1:1000; Santa Cruz Biotechnology, sc-166644), anti-MFN2 (1:1000; Santa Cruz Biotechnology, sc-100560) and anti-β-actin (1:1000; Santa Cruz Biotechnology, sc-47778). The immunocomplexes were then detected using horseradish peroxidase-conjugated IgG (1:1000; Jackson ImmunoResearch Laboratories, PA, USA), and the reaction was developed using an ECL detection kit (Amersham Bioesciences, RPN2108, TN, USA) in an ImageQuant LAS 4000 system (GE Healthcare Life Sciences, MA, USA). Forskolin was used as a positive control treatment for DRP1 S637 phosphorylation. Forskolin activates adenylyl cyclase to increase intracellular cyclic AMP levels, thereby activating PKA, the primary kinase responsible for DRP1 S637 phosphorylation. Forskolin (HY-15371; MedChemExpress, NJ, USA) was added to cells at 10 μM for 30 min before cell lysis. The immunoblots of whole membranes are presented in Fig. S8.

### Time-lapse recording

Time-lapse images of mitochondrial morphology using fluorescent protein-tagged mitochondrial proteins were recorded using an inverted wide-field fluorescence microscope (Olympus IX71, Japan). Cells overexpressing COX8A–mRFP and CatCh–Venus were cultured at a density of 5×10^4^ cells/3 cm in glass-bottomed dishes and maintained in Phenol Red-free medium inside a mini-incubator at 37°C with moderate humidity. Live images of mitochondria were recorded every 5 min for 25 min under blue light illumination. Mitochondrial morphology and state were analyzed using the ImageJ and MicroP software programs, respectively.

### Immunofluorescence staining and imaging

After treatment with 470 nm illumination, COX8A–mRFP-overexpressing U2OS cells were fixed with 4% buffered paraformaldehyde and permeabilized using 0.5% Triton X-100 for 15 min. Cells were stained with rabbit anti-DRP1 antibody (1:100 in PBS; Cell Signaling technology, 8570S) or rabbit anti-phospho-DRP1-S616 antibody (1:100 in PBS; Cell Signaling Technology, 3455) for 12 h at 4°C. In addition, the cells were stained with goat anti-rabbit IgG conjugated to Alexa Fluor 488 secondary antibody (1:200 in PBS; Molecular Probes, 10453272, OR, USA) for 1 h. The fluorophore was excited by a laser at 488 or 543 nm, and fluorescence was detected using a scanning confocal microscope (Olympus FV3000, Japan).

### Assay of mitochondrial functions

ATP synthesis, mitochondrial ROS production, and mitochondrial membrane potential were used to evaluate mitochondrial function. Live-cell staining was used for treatment with specific fluorescent probes, such as ATP-Red 1 (5 μM) to quantify mitochondrial ATP, MitoSOX Red (5 μM) to evaluate mitochondrial ROS, and JC-1 (5 µg/ml) and TMRM (50 nM) to identify the mitochondrial membrane potential. In this study, mitochondrial JC-1 aggregates with red fluorescence emission and JC-1 monomers with green fluorescence emission were separately detected. Confocal microscopy (Olympus FV3000, Japan) and flow cytometry (BD FACSCalibur, CA, USA) were used to capture fluorescence images and quantify fluorescence intensity.

### PI staining for apoptosis detection

To analyze the apoptotic ratio, the cells were fixed in 70% alcohol, treated with 100 mg/ml RNase, and stained with 40 mg/ml propidium iodide (PI; Sigma-Aldrich, P4170). The PI-stained cells were incubated in the dark at room temperature for 30 min and analyzed using flow cytometry (BD FACSCalibur) at an excitation wavelength of 543 nm. The apoptotic ratio was assessed from the hypodiploid DNA peak of apoptotic cells (sub-G1 phase) using the Cell Quest software.

### Live- and dead-cell assay

For cell viability analysis, 5×10^4^ U2OS cells were seeded in 3-cm dishes and grown overnight as a monolayer. The cells were then illuminated with 470 nm blue light with different parameters. Each group of cells was washed with DMEM and stained with 2 μg/ml Hoechst 33342 (nucleus) (Invitrogen, D1306), 1 μM calcein AM (live cells) (Invitrogen, C1430) and 1 μM ethidium homodimer-1 (dead cells) (Invitrogen, E1169) after illumination. Next, fluorescent images were obtained using an inverted wide-field fluorescence microscope (Olympus IX71, Japan), and cell viability was analyzed using the ImageJ software (National Institutes of Health, MD, USA).

### Statistical analysis

All data are reported as the mean±standard error of the mean (s.e.m.) and graphs were generated by OriginPro (OriginLab version 9). Two-tailed paired Student's *t*-test or one-way ANOVA with Dunnett's post hoc test was used to assess the statistically significant differences between the groups. *P<0.05, **P<0.01 and ***P<0.001 were considered to be statistically significant.

## Supplementary Material

10.1242/joces.260819_sup1 Supplementary information
