# Supplementary material for: Optogenetically engineered Ca2+ oscillation-mediated DRP1 activation promotes mitochondrial fission and cell death
Source: J Cell Sci. 2023 Jun 21;136(12):jcs260819. doi: 10.1242/jcs.260819 (PMC10323238; doi:10.1242/jcs.260819)
Supplement: Supplementary information [file joces-136-260819-s1.pdf]

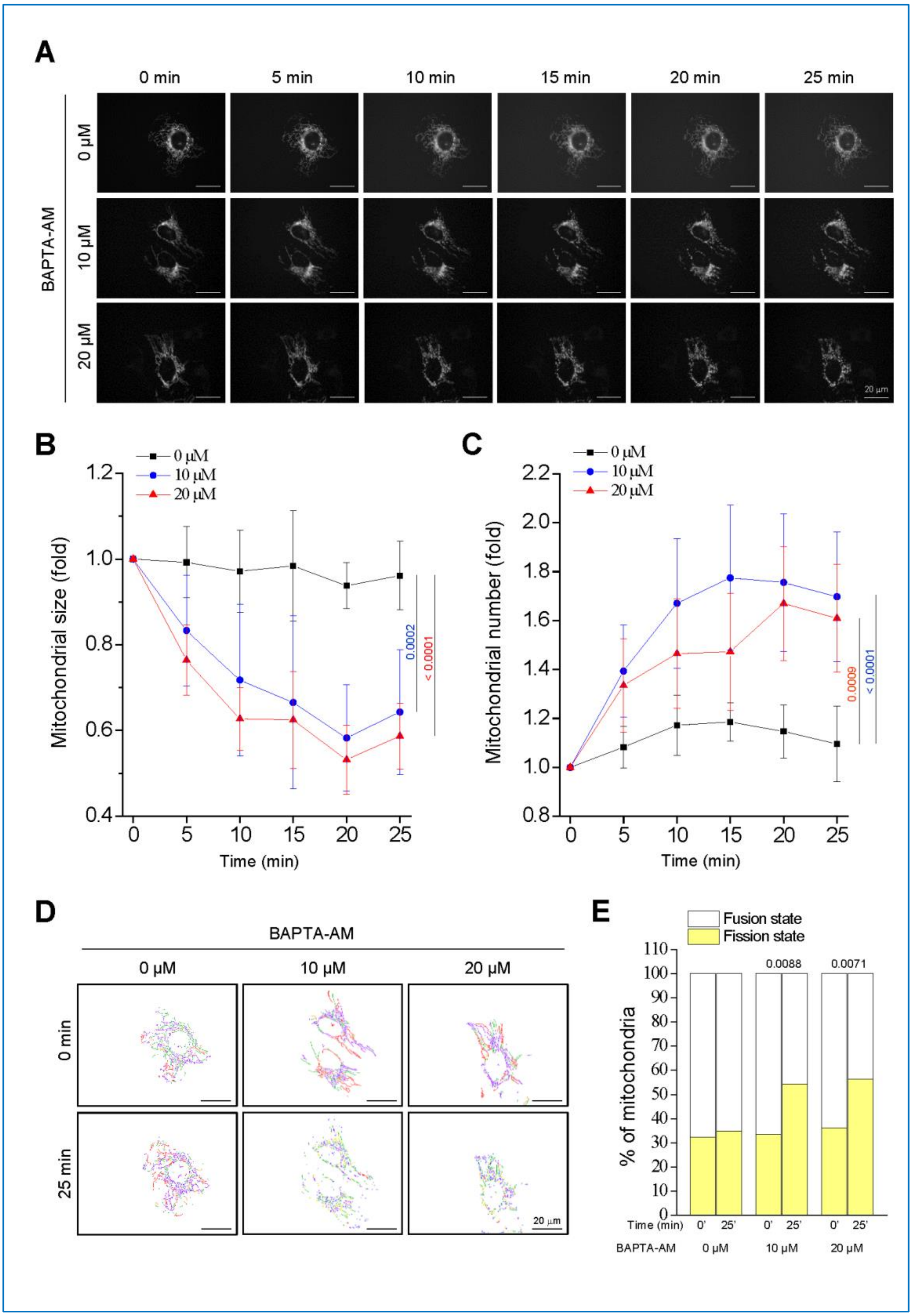

Journal of Cell Science • Supplementary information

Fig. S1. Intracellular Ca^{2+} chelation by BAPTA-AM induces mitochondrial fission. COX8A-mRFP-overexpressing in U2OS cells can be used to monitor the dynamic changes in the morphology of mitochondria. (A) Real-time fluorescence imaging records the mitochondrial fission under BAPTA-AM (0, 10, 20 µM) treatment in 5-min intervals for 25 min. Images shown are representative of three biological repeats. Scale bar, 20 µm. (B,C) Quantitative analysis of mitochondrial (B) average size and (C) number from (A) were calculated using ImageJ at different time points and normalized against its initial state. (D) Images from initial point (0 min) and end point (25 min) under BAPTA-AM (0, 10, 20 µM) treatment of mitochondrial states were classified and the percentage of the different mitochondrial states is presented in Fig. S7B. Scale bar, 20 µm. (E) Statistical analysis of mitochondrial states using the MicroP software. All values are represented as the mean ± SEM from three biological replicates with at least 15 cells per replicate, compared with control group using the one-way ANOVA; adjusted *P*-value is indicated for each comparison.

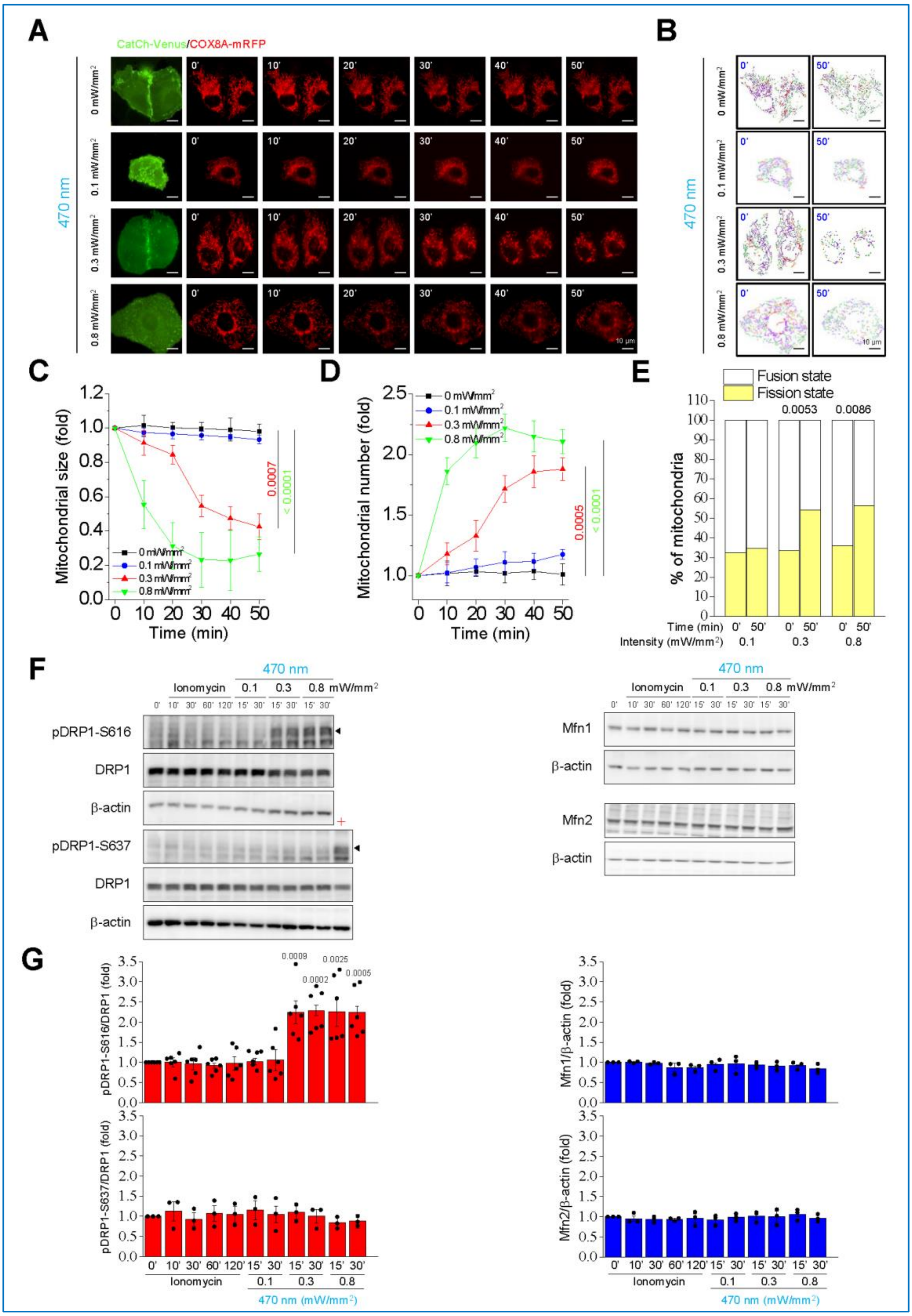
A
CatCh-Venus/COX8A-mRFP
470 nm
0 mW/mm2
0.1 mW/mm2
0.3 mW/mm2
0.8 mW/mm2
0'
10'
20'
30'
40'
50'
10 μm
B
C
Mitochondrial size (fold)
Time (min)
0.0007
< 0.0001
D
Mitochondrial number (fold)
0.0005
< 0.0001
E
Fusion state
Fission state
% of mitochondria
0.0053
0.0086
Time (min)
Intensity (mW/mm2)
F
Ionomycin
pDRP1-S616
DRP1
β-actin
pDRP1-S637
Mfn1
Mfn2
G
pDRP1-S616/DRP1 (fold)
pDRP1-S637/DRP1 (fold)
Mfn1/β-actin (fold)
Mfn2/β-actin (fold)
0.0009
0.0002
0.0025
0.0005
470 nm (mW/mm2)

Fig. S2. Different power intensities of blue light illumination induce Ca^{2+} oscillations to modulate mitochondrial fission. Real-time fluorescence images showed the dynamic changes in the mitochondrial network. The CatCh-Venus and COX8A-mRFP-overexpressing U2OS cells were treated with 470 nm illumination (1 Hz, 250 ms exposure time) at different power intensities (0, 0.1, 0.3, and 0.8 mW/ mm^2) for 50 min. Representative (A) fluorescence and (B) MicroP mitochondrial images and the percentage of the different mitochondrial states is presented in Fig. S7D. Images shown are representative of three biological replicates with at least 15 cells per replicate. Scale bar, 10 µm. (C,D) Quantitative analysis of mitochondrial average (C) size and (D) number from (A) were calculated using ImageJ at different time points and normalized against its initial state. (E) Images from initial point (0 min) and end point (50 min) with light illumination at different power intensities were classified into fusion and fission states. Statistical analysis of mitochondrial states using the MicroP software. All values are represented as the mean ± SEM from three biological replicates with at least 15 cells per replicate, compared with control group using one-way ANOVA; adjusted *P*-value is indicated for each comparison. (F) Western immunoblotting of pDRP1 at S616 (pDRP1-S616) and S637 (pDRP1-S637), DRP1, MFN1, MFN2, and the internal control, β-actin, in whole cell lysates. The forskolin-treated U2OS cells were used as the positive control for phosphorylation of DRP1 at S637 (indicated by +). The immunoblots of the whole membrane are presented on Fig. S8C. (G) Quantification of proteins phosphorylation and expression as relative intensities of the blots are presented as mean ± SEM from three biological replicates calculated using the Student's *t*-test; adjusted *P*-value is indicated for each comparison.

Journal of Cell Science • Supplementary information

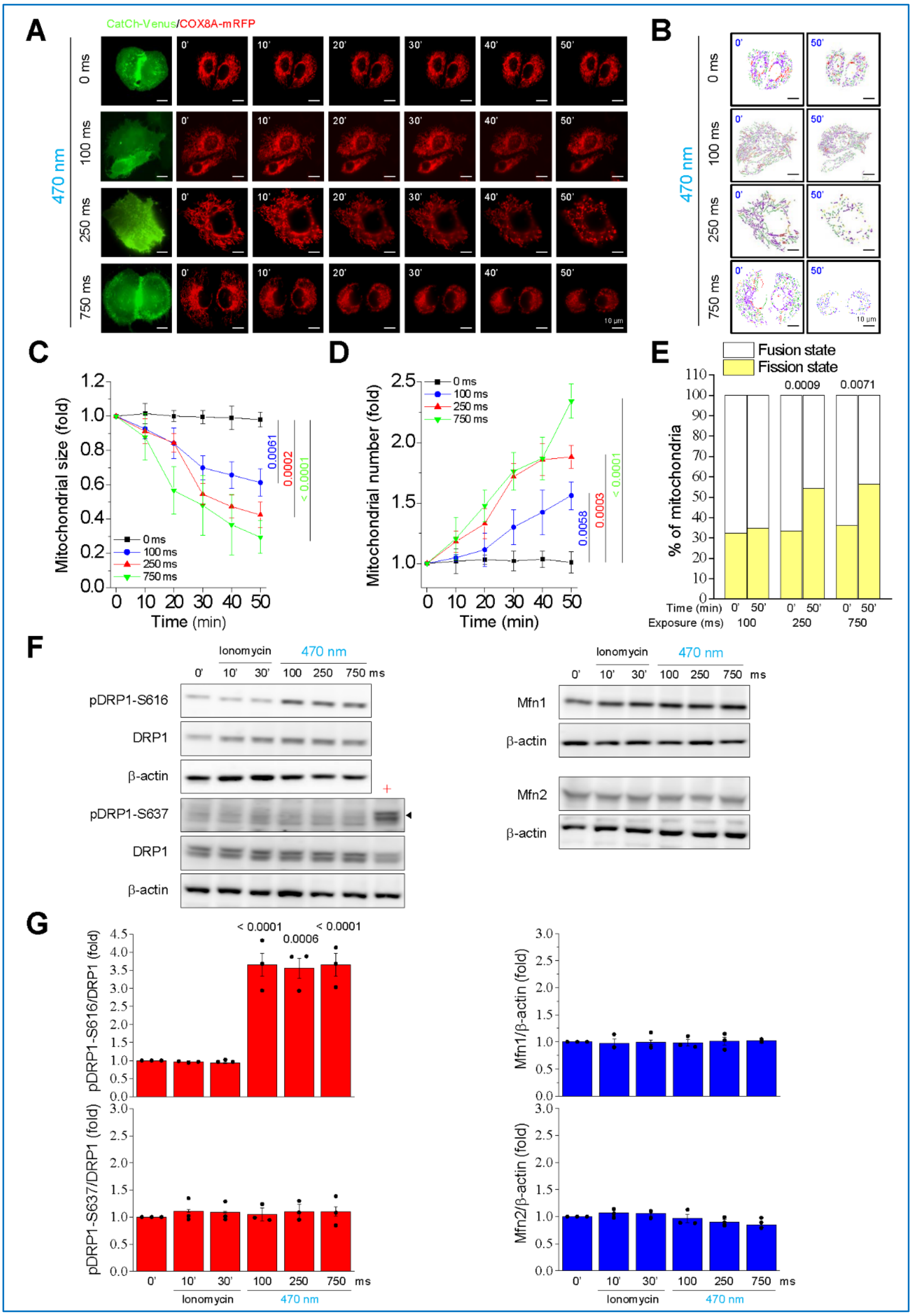

Fig. S3. Different exposure time of blue light illumination induces Ca^{2+} oscillations to modulate mitochondrial fission. Real-time fluorescence images showed the

Journal of Cell Science • Supplementary information

dynamic changes in the mitochondrial network. The CatCh-Venus and COX8A-mRFP-overexpressing U2OS cells were treated with 470 nm illumination (0.3 mW/mm^2, 1 Hz) at different exposure times (0, 100, 250, and 750 ms) for 50 min. Representative (A) fluorescence and (B) MicroP mitochondrial images and the percentage of the different mitochondrial states is presented in Fig. S7E. Images shown are representative of three biological replicates with at least 15 cells per replicate. Scale bar, 10 µm. (C,D) Quantitative analysis of mitochondrial average (C) size and (D) number from (A) using ImageJ at different time points and normalized against its initial state. (E) Images from initial point (0 min) and end point (50 min) with light illumination at different exposure times were classified into fusion and fission states. Statistical analysis of mitochondrial states was counted using the MicroP software. All values are represented as the mean ± SEM from three biological replicates with at least 15 cells per replicate, compared with the control group using one-way ANOVA; adjusted *P*-value is indicated for each comparison. (F) Western immunoblotting of pDRP1 at S616 (pDRP1-S616) and S637 (pDRP1-S637), DRP1, MFN1, MFN2, and the internal control, β-actin, in whole cell lysates. The forskolin-treated U2OS cells were used as the positive control for phosphorylation of DRP1 at S637 (indicated by +). The immunoblots of the whole membrane are presented on Fig. S8D. (G) Quantification of protein phosphorylation and expression as relative intensities of the blots are presented as the mean ± SEM from three biological replicates, calculated using the Student's *t*-test; adjusted *P*-value is indicated for each comparison.

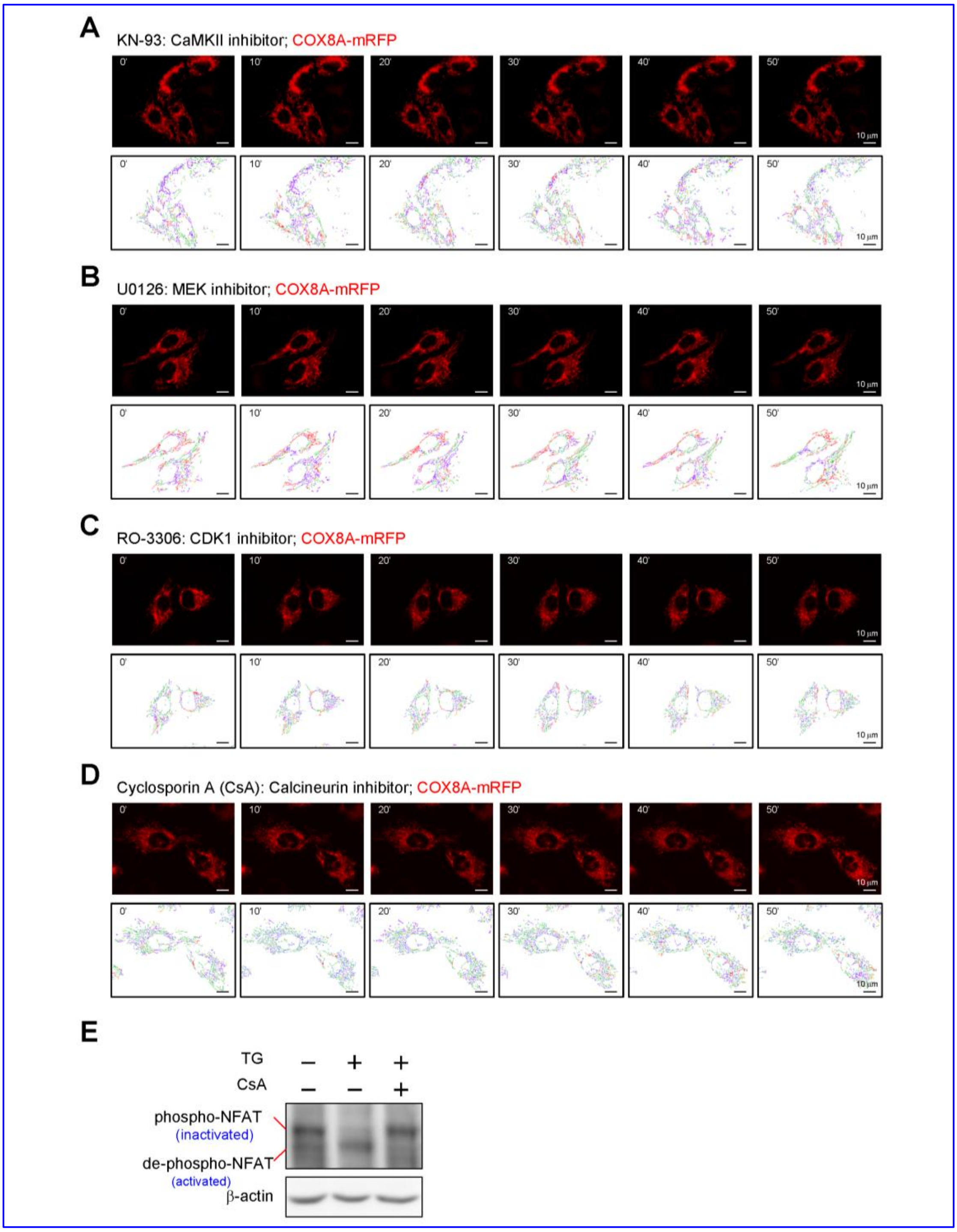

Fig. S4. Inhibitors for upstream inducers of DRP1 activation modulate Ca^{2+}-induced mitochondrial fission. Real-time fluorescence images in 10-min intervals showed the dynamic changes in the mitochondrial network. The CatCh-Venus and COX8A-mRFP-overexpressing U2OS cells were preincubated with (A) 10 µM KN-93 (CaMKII inhibitor), (B) 10 µM U0126 (MEK/ERK inhibitor), (C) 10 µM RO-3306 (CDK1 inhibitor), and (D) 10 µM cyclosporin A (calcineurin inhibitor) for 30 min, and

Journal of Cell Science • Supplementary information

further treated with 470 nm illumination (0.3 mW/mm^2, 0.1 Hz, 250 ms exposure time) for 50 min. Representative mitochondrial fluorescence (upper panel) and MicroP (lower panel) images were chosen from three biological replicates at least 15 cells per replicate. Scale bar, 10 μm. (E) Inhibitory effect of CsA on the nuclear factor of activated T cells (NFAT). Thapsigargin (TG) and CsA were used to induce the activation (dephosphorylation) and inactivation (phosphorylation) of NFAT by modulating calcineurin, respectively. Pretreatment of U2OS cells with or without the calcineurin inhibitor (10 μM CsA) for 30 min, and further treatment with the sarco/endoplasmic reticulum Ca^{2+}-ATPases (SERCA) inhibitor (2 μM TG) for 10 min. Inhibitory effect of CsA on NFAT was tested via immunoblotting. β-actin served as the internal control. The immunoblots of the whole membrane are presented on Fig. S8E.

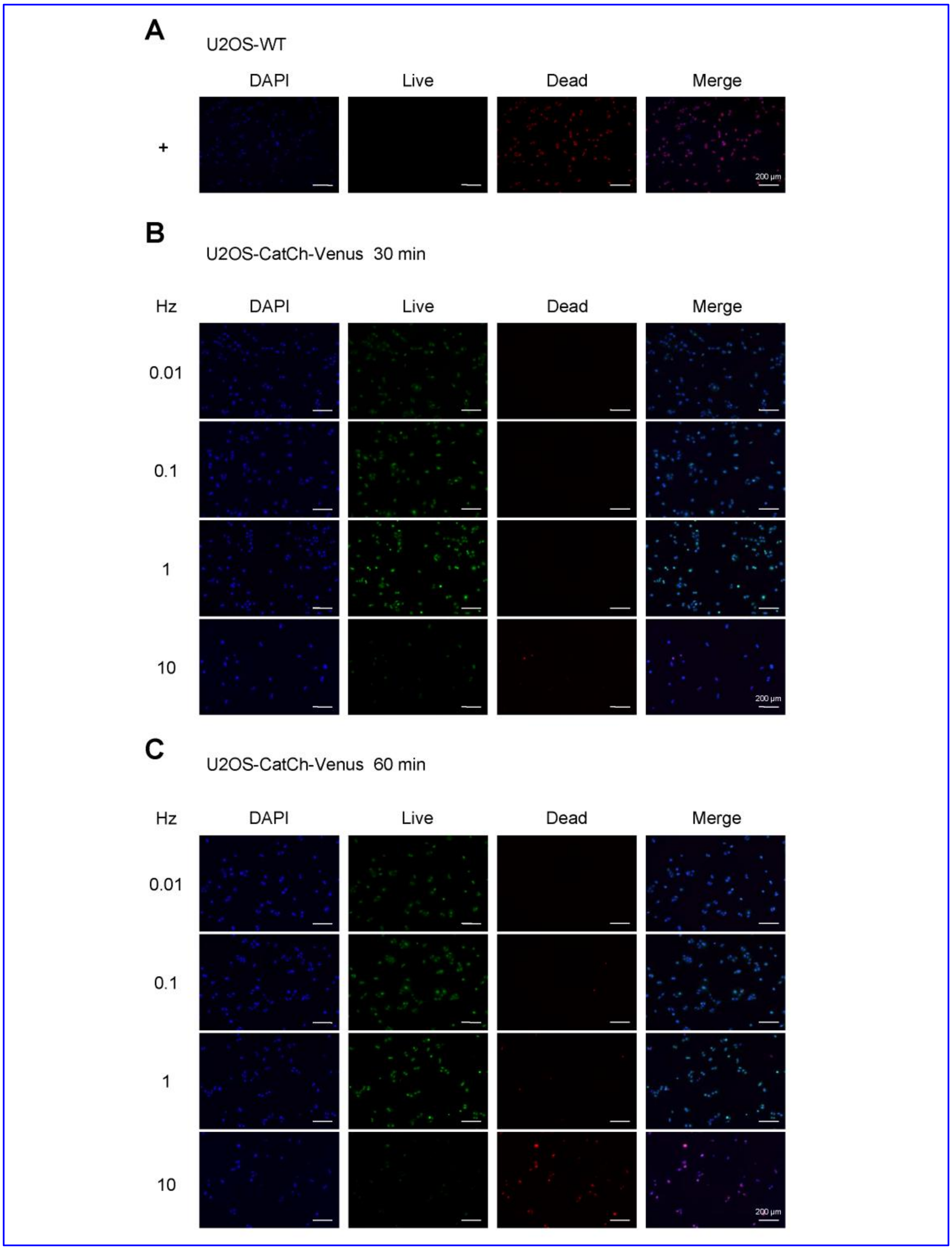

Fig. S5. Effects of CatCh-mediated Ca^{2+} oscillations on cell survival. Live-dead analysis of CatCh-overexpressing U2OS cells (U2OS-CatCh) at 48 h after light illumination. (A) U2OS-WT cells after fixation with 4% paraformaldehyde as a positive control of dead cells. (B,C) Representative fluorescence images (nucleus: blue; live cells: green; dead cells: red) of U2OS-CatCh cells after (B) 30 or (C) 60 min of light illumination at different frequencies (0.01, 0.1, 1, and 10 Hz), fixed 0.1 mW/mm2 power, and 100 ms exposure time. DAPI was used to stain the nuclei. Images shown are representative of three biological replicates. Scale bar, 200 µm.

Journal of Cell Science • Supplementary information

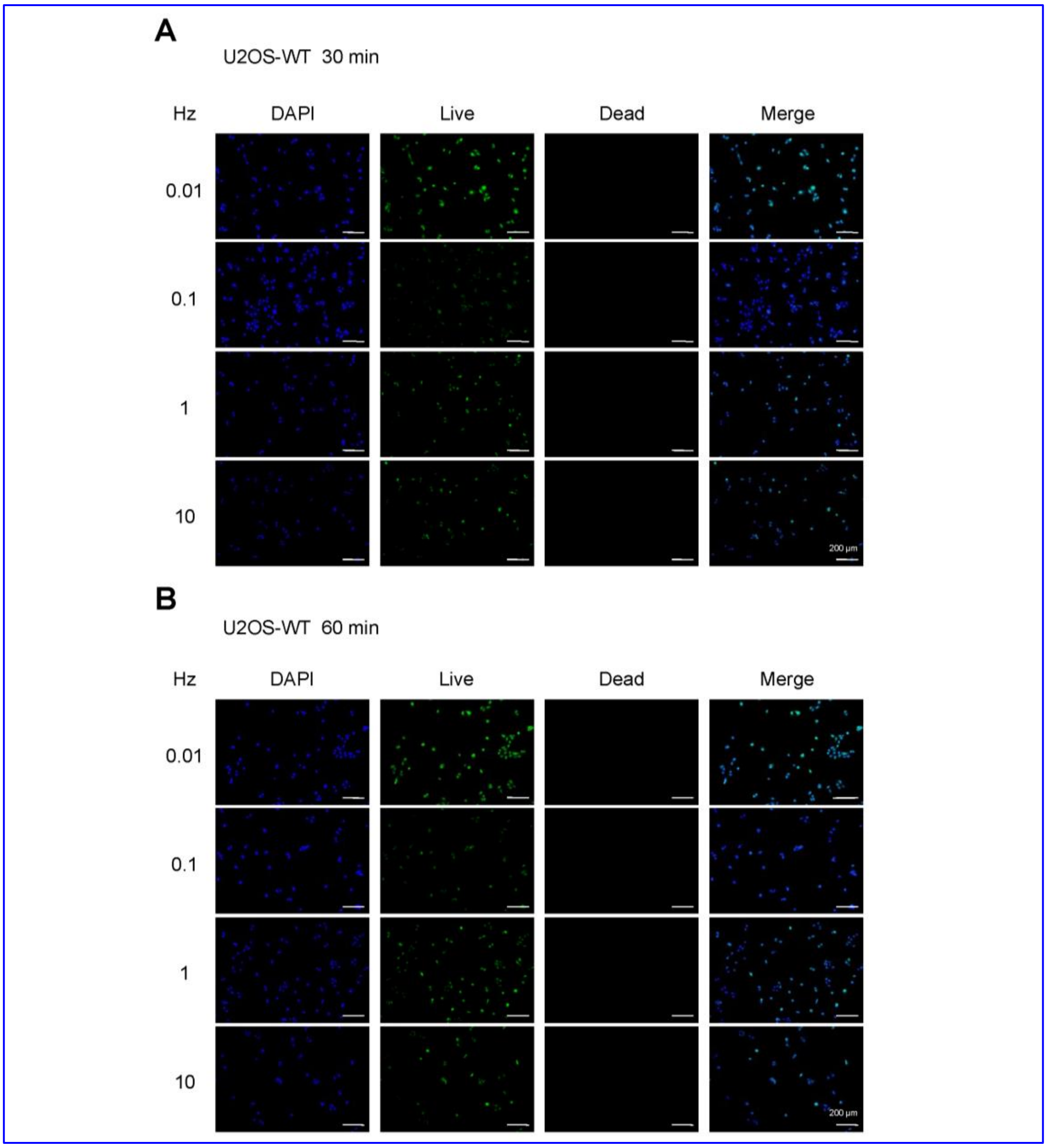

Fig. S6. Effects of light illumination on cell survival. Live-dead analysis of parental U2OS cells (U2OS-WT) at 48 h after light illumination. Representative fluorescence images (nucleus: blue; live cells: green; dead cells: red) of U2OS-WT cells after (A) 30 or (B) 60 min of light illumination at different frequencies (0.01, 0.1, 1, and 10 Hz), fixed 0.1 mW/mm^2 power, and 100 ms exposure time. DAPI was used to stain the nuclei. Images shown are representative of three biological replicates. Scale bar, 200 µm.

Journal of Cell Science • Supplementary information

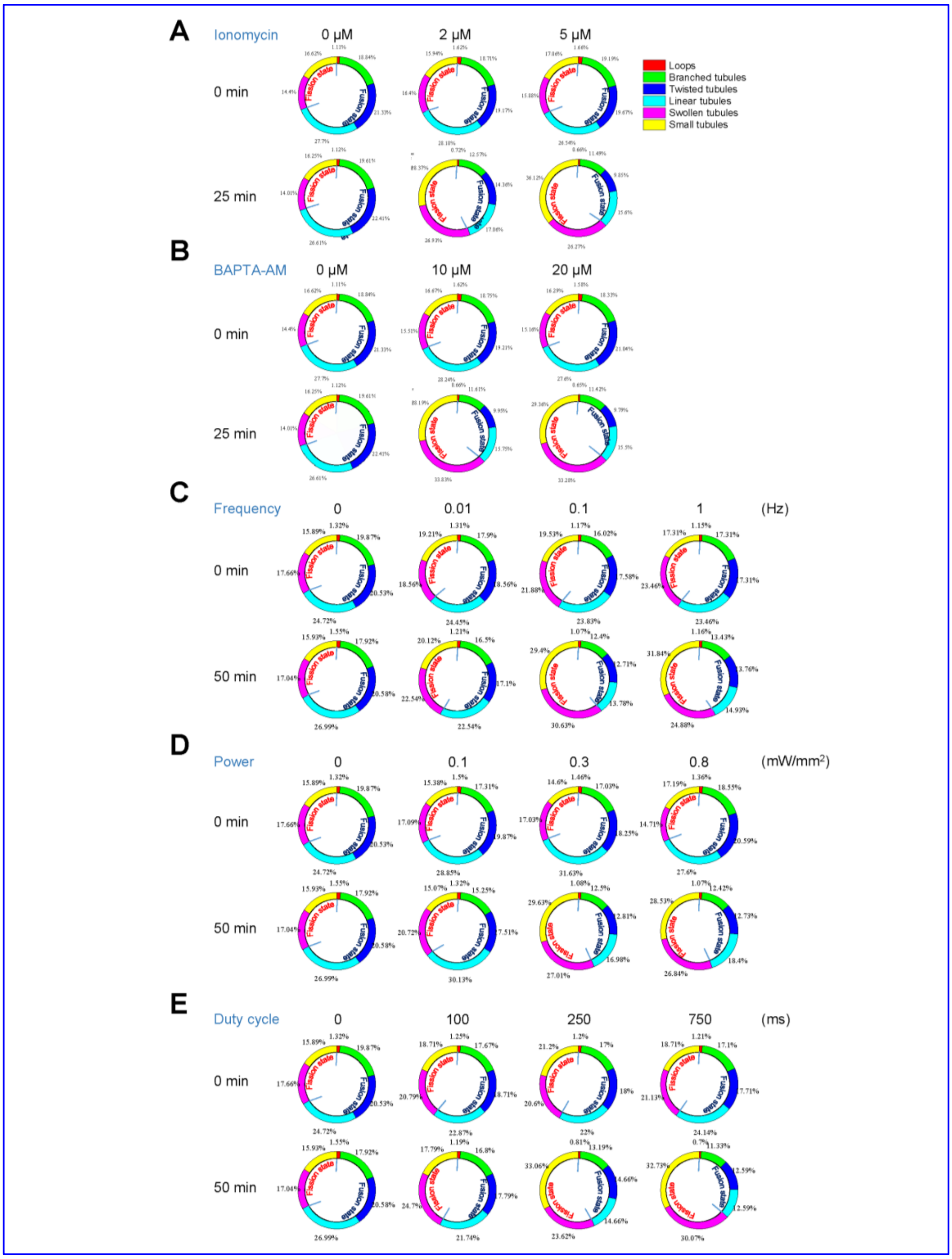

Fig. S7. Percentage of six different mitochondrial states using pie chart. (A) Cytochrome c oxidase subunit 8A (COX8A)-mRFP overexpression in U2OS cells under ionomycin (0, 2, 5 µM) treatment. Quantitative analysis of six different mitochondrial states at the initial point (0 min) and end point (25 min) is presented as a percentage. (B) COX8A-mRFP overexpression in U2OS cells under BAPTA-AM (0,

10, 20 μM) treatment. Quantitative analysis of six different mitochondrial states at the initial point (0 min) and end point (25 min) is presented as a percentage. (C) The CatCh-Venus and COX8A-mRFP-overexpressing U2OS cells were subjected to 470 nm illumination (0.3 mW/mm^2, 250 ms exposure time) at different frequencies (0, 0.01, 0.1, and 1 Hz). Quantitative analysis of six different mitochondrial states at the initial point (0 min) and end point (50 min) is presented as a percentage. (D) The CatCh-Venus and COX8A-mRFP-overexpressing U2OS cells were subjected to 470 nm illumination (0.1 Hz, 250 ms exposure time) at different power intensities (0, 0.1, 0.3, and 0.8 mW/mm^2). Quantitative analysis of six different mitochondrial states at the initial point (0 min) and end point (50 min) is presented as a percentage. (E) The CatCh-Venus and COX8A-mRFP-overexpressing U2OS cells were subjected to 470 nm illumination (0.3 mW/mm^2, 0.1 Hz) at different exposure times (0, 100, 250, and 750 ms). Quantitative analysis of six different mitochondrial states at the initial point (0 min) and end point (50 min) is presented as a percentage. MicroP software was used to quantify the whole set of data above.

Journal of Cell Science • Supplementary information

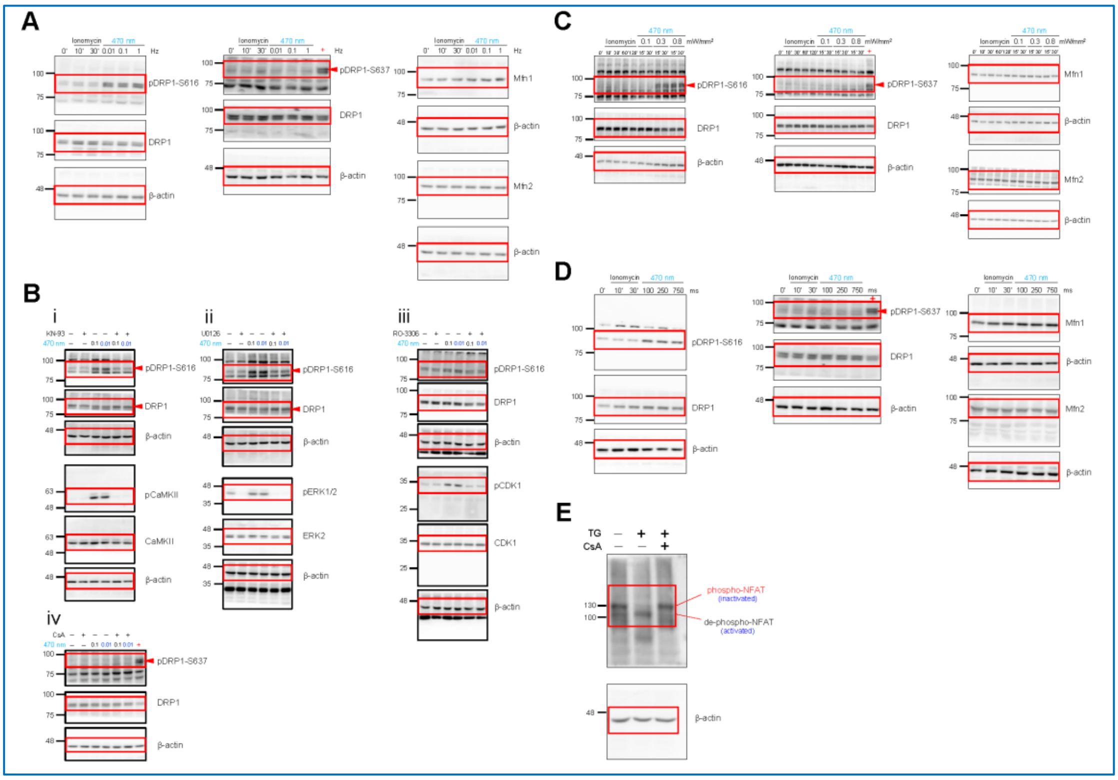

Fig. S8. Blot transparency. (A) The CatCh-Venus and COX8A-mRFP-overexpressing U2OS cells were subjected to 470 nm illumination (0.3 mW/mm^2, 250 ms exposure time) at different frequencies (0, 0.01, 0.1, and 1 Hz) for 50 min. Whole membranes of Western blots were shown to detect phosphorylated dynamin-related protein 1 (DRP1) at serine 616 (pDRP1-S616) and serine 637 (pDRP1-S637), DRP1, mitofusin (MFN)-1, MFN2, and the internal control, β-actin, in whole cell lysates shown in Fig. 3F. The forskolin-treated U2OS cells were used as the positive control for the phosphorylation of DRP1 at S637 (indicated by +). (B) The CatCh-Venus and COX8A-mRFP-overexpressing U2OS cells under pretreatment with or without different inhibitors for 30 min, including 10 µM KN-93, 10 µM U0126, 10 µM RO-3306, and 10 µM cyclosporin A (CsA), followed by treatment with 470 nm illumination (0.3 mW/mm^2, 0.1 Hz, 250 ms exposure time) for 50 min. Whole membrane Western blots were shown to detect pDRP1-S616 and pDRP1-S637, pCaMKII, pERK1/2, pCDKI, DRP1, CaMKII, ERK2, CDK1, and β-actin, in whole cell lysates shown in Fig. 4D,F,H,J, respectively. (C) The CatCh-Venus and COX8A-mRFP-overexpressing U2OS cells were treated with 470 nm illumination (0.1 Hz, 250 ms exposure time) at different power intensities (0, 0.1, 0.3, and 0.8 mW/mm^2) for 50 min. In whole cell lysates shown

Journal of Cell Science • Supplementary information

in Figure S2F, whole membrane Western blots detected pDRP1-S616 and pDRP1-S637, DRP1, MFN1, MFN2, and β-actin, in whole cell lysates presented in Fig. S2F. (D) The CatCh-Venus and COX8A-mRFP-overexpressing U2OS cells were treated with 470 nm illumination (0.3 mW/mm^2, 0.1 Hz) at different exposure times (0, 100, 250, and 750 ms) for 50 min. Whole membrane Western blots were shown to detect pDRP1-S616 and pDRP1-S637, DRP1, MFN1, MFN2, and β-actin, in whole cell lysates presented in Fig. S3F. (E) Thapsigargin (TG) and CsA were used to induce the activation (dephosphorylation) and inactivation (phosphorylation) of NFAT by modulating calcineurin, respectively. Pretreatment of U2OS cells with or without the calcineurin inhibitor (10 μM CsA) for 30 min, and further treatment with the SERCA inhibitor (2 μM TG) for 10 min. Whole membrane immunoblotting was shown to test inhibitory effect of CsA on NFAT, β-actin served as the internal control, in whole cell lysates presented in Fig. S4E. KN-93, CaMKII inhibitor; U0126, MEK1/2 inhibitor; RO-3306, CDK1 inhibitor; CsA, calcineurin inhibitor.

Journal of Cell Science • Supplementary information

Table S1. Antibodies, chemical reagents, plasmids and software.

Reagent or Resource	Source	Identifier (Cat No.)
Antibodies		
Mouse anti-NFATC1	Invitrogen	MA3-024
Rabbit anti-LC3A/LC3B	Invitrogen	PA1-16931
Rabbit anti-ERK1 + ERK2 phospho (Thr202/Tyr204)	arigo Biolaboratories	ARG52277
Mouse anti-ERK1 + ERK2	arigo Biolaboratories	ARG62350
Rabbit anti-CaMKII	ABclonal	A2508
Rabbit anti-Phospho-DRP1 (Ser616)	Cell Signaling	3455
Rabbit anti-Phospho-DRP1 (Ser637) (D3A4)	Cell Signaling	6319
Rabbit anti-Phospho-CaMKII (Thr286) (D21E4)	Cell Signaling	12716
Mouse anti-Mfn1	Santa Cruz	sc-166644
Mouse anti-Mfn2	Santa Cruz	sc-100560
Mouse anti-β-actin	Santa Cruz	sc-47778
Peroxidase AffiniPure Goat Anti-Mouse IgG (H+L)	Jackson ImmunoResearch	115-035-003
Peroxidase AffiniPure Goat Anti Rabbit IgG (H+L)	Jackson ImmunoResearch	111-035-003
Alexa Fluor-488 anti-rabbit IgG	Molecular Probes	10453272
Chemical reagents		
U0126	Sigma-Aldrich	662005
Cyclosporin A	Sigma-Aldrich	30024
ATP-Red 1	Sigma-Aldrich	SCT045
Thapsigargin	Sigma-Aldrich	T9033
Ionomycin	Sigma-Aldrich	I0634
BAPTA-AM	Sigma-Aldrich	A1076
KCN	Sigma-Aldrich	60178
Propidium iodide	Sigma-Aldrich	P4170
MitoSOX Red	Invitrogen	M36008
Tetramethylrhodamine, methyl ester (TMRM)	Invitrogen	T668
4’,6-diamidino-2-phenylindole dihydrochloride (DAPI)	Invitrogen	D1306
Calcein AM	Invitrogen	C1430
Ethidium-1	Invitrogen	E1169
KN-93	Cayman	13319
3-MA	Cayman	13242
JC-1	BioVision	1130
RO-3306	Santa Cruz	872573-93-8
Plasmids		
Venus-tagged CatCh (CatCh-Venus)	Takeharu Nagai	Osaka University
mRFP-tagged cytochrome c oxidase subunit 8A (COX8A-mRFP)	Muniswamy Madesh	UT Health, San Antonio
GFP-tagged porin (Mito-GFP)	Addgene	174180
LAR-GECO1.2-mt	LumiSTAR	AL002a
R-GECO	Addgene	32444
Others		
Low-glucose Dulbecco’s modified Eagle’s medium	GIBCO	12491015
Fetal bovine serum	GIBCO	10437028
Penicillin-Streptomycin	Simply	CC502-0100
Plasmid DNA extraction kit	Favorgen	FAPDE 005C1-020
Lipofectamine 3000	Invitrogen	L3000015
G418	GIBCO	11811098
Software		
TILLvisION 4.0	Till Photonics	https://tillvision.software.informer.com/
ImageJ	NIH	https://imagej.nih.gov/nih-image/
MicroP	Biomedical Image Informatics Lab	http://bmi.ym.edu.tw/jypeng/

Journal of Cell Science • Supplementary information

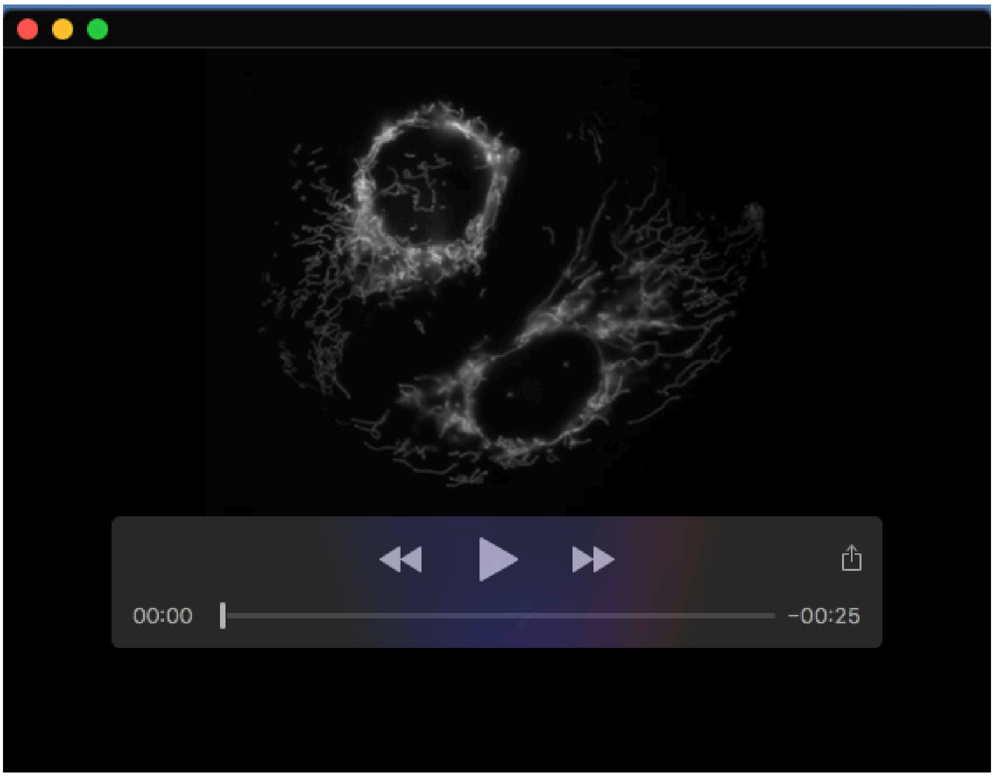

Movie 1. Time-lapse recording of mitochondrial dynamics.

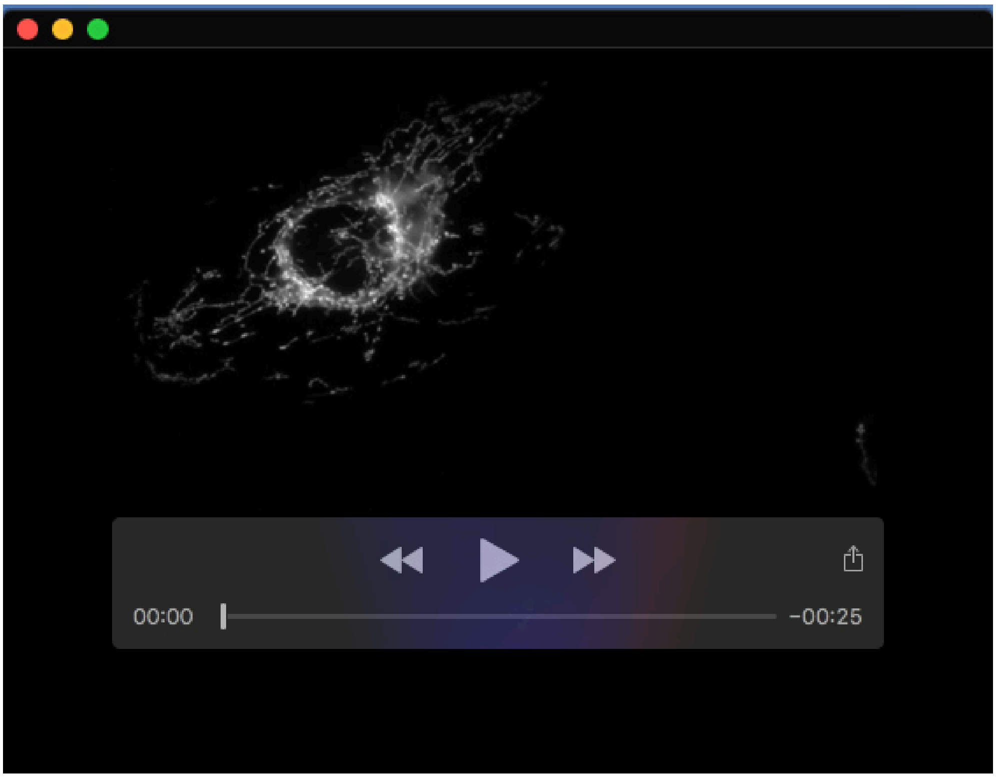

Movie 2. Time-lapse recording of ionomycin-induced mitochondrial fission.

Journal of Cell Science • Supplementary information

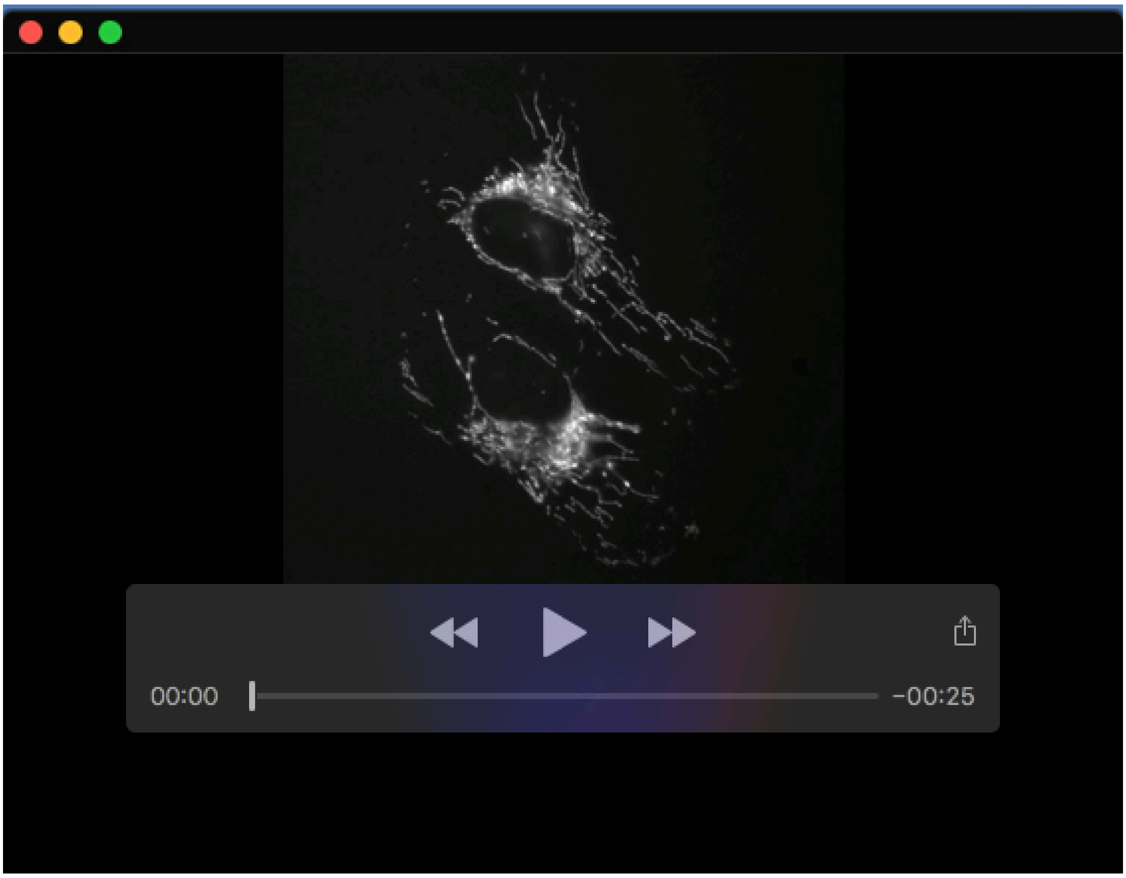

Movie 3. Time-lapse recording of BAPTA-AM induced mitochondrial fission.

Journal of Cell Science • Supplementary information
