# Supplementary material for: Optogenetically engineered Ca2+ oscillation-mediated DRP1 activation promotes mitochondrial fission and cell death
Source: J Cell Sci. 2023 Jun 21;136(12):jcs260819. doi: 10.1242/jcs.260819 (PMC10323238; doi:10.1242/jcs.260819)
Supplement: Supplementary file 1 [file joces-136-260819_review_history.pdf]

Optogenetically engineered Ca^{2+} oscillation-mediated DRP1 activation promotes mitochondrial fission and cell death

Yi-Shyun Lai, Cheng-Chi Chang, Yong-Yi Chen, Thi My Hang Nguyen, Jixuan Xu, Ying-Chi Chen, Yu-Fen Chang, Chia-Yih Wang, Pai-Sheng Chen, Shih-Chieh Lin, I-Chen Peng, Shaw-Jenq Tsai and Wen-Tai Chiu
DOI: 10.1242/jcs.260819

Editor: Michael Way

Review timeline
Original submission: 20 November 2022
Editorial decision: 7 February 2023
First revision received: 14 April 2023
Editorial decision: 3 May 2023
Second revision received: 4 May 2023
Accepted: 5 May 2023

Original submission

First decision letter

MS ID#: JOCES/2022/260819

MS TITLE: Optogenetically engineered Ca^{2+} oscillation-mediated DRP1 activation promotes mitochondrial fission and cell death

AUTHORS: Wen-Tai Chiu, Yi-Shyun Lai, Cheng-Chi Chang, Yong-Yi Chen, Jixuan Xu, Ying-Chi Chen, Yu-Fen Chang, Chia-Yih Wang, Pai-Sheng Chen, Shih-Chieh Lin, I-Chen Peng, and Shaw-Jenq Tsai
ARTICLE TYPE: Research Article

We have now reached a decision on the above manuscript.

To see the reviewers' reports and a copy of this decision letter, please go to: https://submit-jcs.biologists.organd click on the 'Manuscripts with Decisions' queue in the Author Area. (Corresponding author only has access to reviews.)

As you will see, the reviewers a positive but raise a number of criticisms and questions that prevent me from accepting the paper at this stage. They suggest, however, that a revised version might prove acceptable, if you can address their concerns with additional experiments where necessary. If you think that you can deal satisfactorily with the criticisms on revision, I would be pleased to see a revised manuscript.

Please ensure that you clearly highlight all changes made in the revised manuscript. Please avoid using 'Tracked changes' in Word files as these are lost in PDF conversion.

I should be grateful if you would also provide a point-by-point response detailing how you have dealt with the points raised by the reviewers in the 'Response to Reviewers' box. Please attend to all of the reviewers' comments. If you do not agree with any of their criticisms or suggestions please explain clearly why this is so.

© 2023. Published by The Company of Biologists under the terms of the Creative Commons Attribution License (https://creativecommons.org/licenses/by/4.0/).

Reviewer 1

Advance summary and potential significance to field

Lai et al. present a research article describing the use of optogenetics (specifically, Ca^{2+}-translocating channelrhodopsin) to alter intracellular Ca^{2+} signalling, and examining the impact of such Ca^{2+} oscillations on mitochondrial dynamics, specifically fission. By using this approach, they show that optogenetically-driven Ca^{2+} oscillations could promote mitochondrial fission by increasing phosphorylation at Ser616 of DRP1 (via CaMKii, ERK and CDK1), but not by altering phosphorylation at S637 of DRP1. Furthermore, Ca^{2+} oscillations could increase cell death (potentially via apoptosis) and LDH cytotoxicity. This optogentic approach has the potential to provide more flexibility than pharmacological approaches to altering Ca^{2+} signalling, in particular by enabling much finer temporal (and potentially spatial) precision.

The main strength of this manuscript is showing that Ca^{2+} oscillations can be varied using different laser powers and frequency of stimulation bursts to have a tunable impact on mitochondrial fission.

Comments for the author

1. The introduction would benefit from a broader discussion of intracellular Ca^{2+} oscillations, and how the optogenetic method described in this paper is advantageous over pharmacological methods for altering intracellular Ca^{2+} levels.

2. Figure 1a and f could be improved by including zoomed in images to more clearly demonstrate the quoted changes in morphology of mitochondria.

3. Figure 3: Why is there no change in pDrp1-S616 with Ionomycin?

4. Figure 4: For clarity, please state what the inhibitors inhibit.

5. Figure 7: H and I show increase in LC3 puncta, blocked by 3-MA. The authors mention mitophagy here and p23 line 19, but they are not specifically detecting mitophagy in these experiments. Mitophagy experiments should be included (e.g. using mito-Keima, mito-SRAI, Parkin translocation to mitochondria and/or PINK1-dependent phosphorylation of ubiquitin at Serine 65 as dedicated readouts of mitophagy), or the text should be clarified to reflect what is being shown in the experiments.

6. The authors report that their optogenetic method has increased spatial control, but I didn’t see experiments that demonstrate this. For example, can it be used to selectively stimulate specific cells? If not, then the claim of increased spatial control should be omitted.

Additional minor comments
1. Some typographical/grammatical errors to be corrected e.g. p11 lines 4-5, 8-9
p14, lines 10-13
p32, line 3

2. Please could all individual data points be shown in graphs to give a better idea of data spread, and actual p-values given in figure legends.

3. The display of microscopy images could be improved by using colour-blind accessible colours.

Reviewer 2

Advance summary and potential significance to field

This paper provides the useful tool to study the effects of calcium oscillations on mitochondrial function and morphology. Taking into account the importance of calcium signaling in the cell function this methodology is of potential significance to the field.

© 2023. Published by The Company of Biologists under the terms of the Creative Commons Attribution License (https://creativecommons.org/licenses/by/4.0/).

Comments for the author

Manuscript is well-written and experimental design is good. I have several suggestions:
1. The inclusion of the ionomycin part is not necessary it doesn't seem to add to the main topic of the study.
2. I assume that CSA tests are for exploration of the role of mPTP. This is not stated explicitly. Some more explanations/discussion is needed.
3. Ideally authors should include experiments with TMRM to address the issue of membrane potential changes as JC-1 probe is not optimal for these measurements.

First revision

Author response to reviewers' comments

We really appreciate the editor and reviewers for reviewing this manuscript and providing valuable suggestions and comments. We are grateful for giving us the opportunity to revise our manuscript. The additional modifications have been provided as shown in the revised manuscript.

Editor (Comments to the Author):

Dear Dr. Chiu,
We have now reached a decision on the above manuscript.
To see the reviewers' reports and a copy of this decision letter, please go to: https://submit-jcs.biologists.org and click on the 'Manuscripts with Decisions' queue in the Author Area. (Corresponding authors only have access to reviews.)

As you will see, the reviewers are positive but raise a number of criticisms and questions that prevent me from accepting the paper at this stage. They suggest, however, that a revised version might prove acceptable, if you can address their concerns with additional experiments where necessary. If you think that you can deal satisfactorily with the criticisms on revision, I would be pleased to see a revised manuscript.

Please ensure that you clearly highlight all changes made in the revised manuscript. Please avoid using 'Tracked changes' in Word files as these are lost in PDF conversion.

I should be grateful if you would also provide a point-by-point response detailing how you have dealt with the points raised by the reviewers in the 'Response to Reviewers' box. Please attend to all of the reviewers' comments. If you do not agree with any of their criticisms or suggestions please explain clearly why this is so.

Journal of Cell Science is committed to maintaining the integrity of the scientific record. Please read our requirements for preparing your figures (jcs_revision.pdf, attached) to avoid a potential delay in the publication process or rejection on the basis of non- compliance with these guidelines. This guide also includes recommendations on improving figure layout to help reviewers and readers appreciate your data.

For best practice and transparency, and to allow better assessment of the quality of the data and whether the data support the conclusions, we strongly recommend that you:
1. Use graphs that allow the reader to see the true data spread (i.e. box and whisker plots, super plots, etc. See PMID: 32346721 for more information.). If using box-and- whisker plots, please also state what the whiskers represent. See here for helpful information.
Response:
Thank you for the assistance and guidance that you have provided us in better revising our manuscript. We have added the true data spread to the quantitative data in our revised figures.

2. Use appropriate statistics with the sample size representing biological replicates rather than technical replicates.

© 2023. Published by The Company of Biologists under the terms of the Creative Commons Attribution License (https://creativecommons.org/licenses/by/4.0/).

Response:
We appreciate your time and consideration of our manuscript. In the previous submission, our data represented biological replicates rather than technical replicates. However, we usually mention that our data are shown in three independent experiments.

Our previous submission showed the mean ± SEM from at least three independent experiments, but it did not include individual data points. Therefore, we added all individual data points in the revised manuscript.

3. Provide, if using western blots, one additional supplementary figure (as a single PDF or TIFF file). It will not contribute towards your supplementary figure limit. If you provided this at submission and named it 'Blot transparency', please now number this supplementary figure and combine it with the rest of the supplementary material pdf. Please ensure that you cite this figure in your Materials & Methods section, ideally in the western blots paragraph.
Response:
Thank you for taking the time to provide us with such detailed guidance. We have presented the whole membrane of the Western blots in a previous submission. However, we did not combine these supplementary figures into only one additional supplementary figure. Therefore, we reconstructed, named, and combined these whole membranes of the Western blot into one additional Fig. S8 with the rest of the supplementary material PDF file.

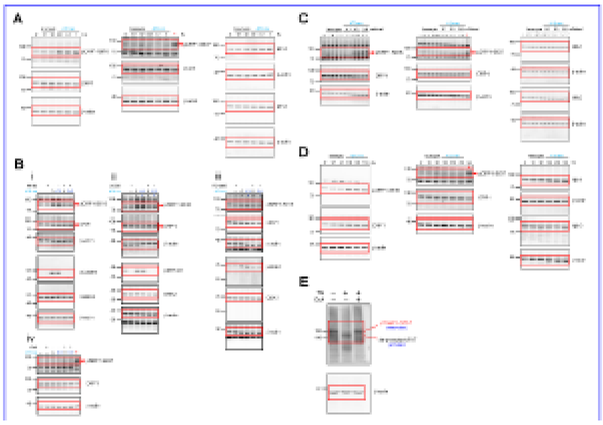

We have added this figure as “Fig. S8” in the supplementary information.

FIGURE S8
Fig. S8. Blot transparency.
(A) The CatCh-Venus and COX8A-mRFP-overexpressing U2OS cells were subjected to 470 nm illumination (0.3 mW/mm^2, 250 ms exposure time) at different frequencies (0, 0.01, 0.1, and 1 Hz) for 50 min. Whole membranes of Western blots were shown to detect phosphorylated dynamin-related protein 1 (DRP1) at serine 616 (pDRP1-S616) and serine 637 (pDRP1-S637), DRP1, mitofusin (MFN)-1, MFN2, and the internal control, ß-actin, in whole cell lysates shown in Fig. 3F. The forskolin-treated U2OS cells were used as the positive control for the phosphorylation of DRP1 at S637 (indicated by +). **(B)** The CatCh-Venus and COX8A-mRFP-overexpressing U2OS cells under pretreatment with or without different inhibitors for 30 min, including 10 μM KN-93, 10 μM U0126, 10 μM RO-3306, and 10 μM cyclosporin A (CsA), followed by treatment with 470 nm illumination (0.3 mW/mm^2, 0.1 Hz, 250 ms exposure time) for 50 min. Whole membrane Western blots were shown to detect pDRP1-S616 and pDRP1-S637, pCaMKII, pERK1/2, pCDKI, DRP1, CaMKII, ERK2, CDK1, and ß- actin, in whole cell lysates shown in Fig. 4D,F,H,J, respectively. **(C)** The CatCh-Venus and COX8A-mRFP-overexpressing U2OS cells were treated with 470 nm illumination (0.1 Hz, 250 ms exposure time) at different power intensities (0, 0.1, 0.3, and 0.8 mW/mm^2) for 50 min. In whole cell lysates shown in Fig. S2F, whole membrane Western blots detected pDRP1-S616 and pDRP1-S637, DRP1, MFN1, MFN2, and ß- actin. **(D)** The CatCh-Venus and COX8A-mRFP-overexpressing U2OS cells were treated with 470 nm illumination (0.3 mW/mm^2, 0.1 Hz) at different exposure times (0,100, 250, and 750 ms) for 50 min. Whole membrane Western blots were shown to detect

© 2023. Published by The Company of Biologists under the terms of the Creative Commons Attribution License (https://creativecommons.org/licenses/by/4.0/).

pDRP1-S616 and pDRP1-S637, DRP1, MFN1, MFN2, and ß-actin, in whole cell lysates presented in Fig. S3F. **(E)** Thapsigargin (TG) and CsA were used to induce the activation (dephosphorylation) and inactivation (phosphorylation) of NFAT by modulating calcineurin, respectively. Pretreatment of U2OS cells with or without calcineurin inhibitor (10 μM CsA) for 30 min, and further treatment with SERCA inhibitor (2 μM TG) for 10 min. Whole membrane immunoblotting was shown to test the inhibitory effect of CsA on NFAT, ß-actin served as the internal control, in whole cell lysates presented in Figure S4E. KN-93, CaMKII inhibitor; U0126, MEK1/2 inhibitor; RO-3306, CDK1 inhibitor; CsA, calcineurin inhibitor.

We thank the reviewer for reviewing this manuscript to provide valuable suggestions and comments. The additional modifications have been provided as shown in the revised manuscript.

Reviewer #1 (Comments to the Author):

Lai et al. present a research article describing the use of optogenetics (specifically, Ca^{2+}-translocating channelrhodopsin) to alter intracellular Ca^{2+} signalling, and examining the impact of such Ca^{2+} oscillations on mitochondrial dynamics, specifically fission. By using this approach, they show that optogenetically-driven Ca^{2+} oscillations could promote mitochondrial fission by increasing phosphorylation at Ser616 of DRP1 (via CaMKII, ERK and CDK1), but not by altering phosphorylation at S637 of DRP1. Furthermore, Ca^{2+} oscillations could increase cell death (potentially via apoptosis) and LDH cytotoxicity. This optogentic approach has the potential to provide more flexibility than pharmacological approaches to altering Ca^{2+} signalling, in particular by enabling much finer temporal (and potentially spatial) precision.

The main strength of this manuscript is showing that Ca^{2+} oscillations can be varied using different laser powers and frequency of stimulation bursts to have a tunable impact on mitochondrial fission.

1. The introduction would benefit from a broader discussion of intracellular Ca^{2+} oscillations, and how the optogenetic method described in this paper is advantageous over pharmacological methods for altering intracellular Ca^{2+} levels.

Response:

Thank you for your constructive suggestion.

Ca^{2+} oscillation is a pervasive signal present in all cells that provides an effective way to transmit intracellular biological information; for example, Ca^{2+} oscillation controls the release of neurotransmitter in neuron cells (Fu et al., 2008), the contraction of smooth muscle cells in pulmonary and vascular vessels (Pauvert et al., 2000; Perez and Sanderson, 2005; Fan et al., 2007), gene expression and cell differentiation (Yildirim and Bertram, 2017; Okada et al., 2020). Ca^{2+} oscillation has been reported to be related to the activation of intracellular signaling, such as the regulation of intracellular transcription factors (Lai et al., 2021) and mitochondrial metabolism (Wacquier et al., 2016). Moreover, a number of direct and indirect mechanisms regulate the amplitude, frequency, and duty cycle of intracellular Ca^{2+} oscillations (Parekh, 2011; Smedler and Uhlén, 2014).

*We have added this part to (**page 5 and line 8 to line 18**) of the "Introduction" section of the revised manuscript for "a broader discussion of intracellular Ca^{2+} oscillations."*

Optogenetic manipulation actively controls Ca^{2+} signals in ways that cannot be achieved by pharmacological methods, which includes frequency, amplitude, duty cycle and work duration. Through pharmacological approaches, Ca^{2+} signal can only be recorded passively (Carter and de Lecea, 2011; Lai et al., 2021).

© 2023. Published by The Company of Biologists under the terms of the Creative Commons Attribution License (https://creativecommons.org/licenses/by/4.0/).

*We have added this part to (**page 9 and line 5 to line 9**) the "Introduction" section of the revised manuscript for "how the optogenetic method is advantageous over pharmacological methods for altering intracellular Ca^{2+}"*

We have added below references to the "References" section of the revised manuscript.

Carter, M. E. and de Lecea, L. (2011). Optogenetic investigation of neural circuits in vivo. *Trends Mol. Med.* **17**, 197-206.
Fan, C., Su, Q., Li, Y., Guggino, W. B. and Johns, R. A. (2007). Intracellular calcium oscillations induced by hypoxia induced mitogenic factor in pulmonary artery smooth muscle cells. *FASEB J.* **21**, A1351-A1351.
Fu, M., Sun, Z. H., Zong, M., He, X. P., Zuo, H. C. and Xie, Z. P. (2008). Deoxyschisandrin modulates synchronized Ca^{2+} oscillations and spontaneous synaptic transmission of cultured hippocampal neurons. *Acta Pharmacol. Sin.* **29**, 891-898.
Okada, H., Okabe, K. and Tanaka, S. (2020). Finely-tuned calcium oscillations in osteoclast differentiation and bone resorption. *Int. J. Mol. Sci.* **22**, 180.
Pauvert O., Marthan R. and Savineau J. (2000). NO-induced modulation of calcium- oscillations in pulmonary vascular smooth muscle. *Cell Calcium* **27**, 329-338.
Perez, J. F. and Sanderson, M. J. (2005). The contraction of smooth muscle cells of intrapulmonary arterioles is determined by the frequency of Ca^{2+} oscillations induced by 5-HT and KCl. *J. Gen. Physiol.* **125**, 555-567.
Wacquier, B., Combettes, L., Van Nhieu, G. T. and Dupont, G. (2016). Interplay between intracellular Ca^{2+} oscillations and Ca^{2+}-stimulated mitochondrial metabolism. *Sci. Rep.* **6**, 19316.
Yildirim, V. and Bertram, R. (2017). Calcium oscillation frequency-sensitive gene regulation and homeostatic compensation in pancreatic β-Cells. *Bull. Math. Biol.* **79**, 1295-1324.

2. Figure 1a and f could be improved by including zoomed in images to more clearly demonstrate the quoted changes in morphology of mitochondria.

Response:

We truly appreciate your advice. In our previous submission, the images were too small to observe mitochondrial fission over time. Therefore, we have enlarged Fig. 1A, F to be more distinguishable.

3. Figure 3: Why is there no change in pDRP1-S616 with Ionomycin?

Response:

Thank you so much for pointing out the important question. Ca^{2+} signaling is one of the most versatile and universal signaling agents to convert extracellular and intracellular information into specific cellular outcomes, including proliferation, differentiation, muscle contraction, learning and cell death. The interpretation of an increase in intracellular Ca^{2+} signal requires consideration of different properties, such as amplitude, mean concentration, frequency and also the wave speed as mechanisms for signaling (Palk et al., 2012). The cytosolic Ca^{2+} concentration can start oscillating as a result of the stimulation or complex coordinated activity involving many Ca^{2+} transporters in the cell. Ca^{2+} oscillation occurs through amplitude modulation (AM) and frequency modulation (FM) (Samanta and Parekh, 2017). Indeed, optogenetics-induced Ca^{2+} signal belongs to Ca^{2+} oscillations.

In our results, increasing Ca^{2+} concentrations induced a response in the AM of Ca^{2+} oscillations (**Rebuttal Fig. 1A**). In contrast, Ca^{2+} oscillations are decoded by FM, which responds to change in oscillation frequency but not Ca^{2+} concentration (**Rebuttal Fig. 1B**). In addition, different frequencies of intracellular Ca^{2+} oscillating waves seem to be the most common strategy used by the cell to transmit Ca^{2+} messages under many different physiological stimuli (Lai et al., 2021).

Ionomycin is an effective Ca^{2+} ionophore that is commonly used to modify intracellular Ca^{2+} concentrations. It can increase and sustain Ca^{2+} concentration, called "steady-state Ca^{2+}" (leaving the initial peak Ca^{2+} response unaffected but not Ca^{2+} oscillation (**Rebuttal Fig. 1C**). In

© 2023. Published by The Company of Biologists under the terms of the Creative Commons Attribution License (https://creativecommons.org/licenses/by/4.0/).

our previous study, we found that low frequency Ca^{2+} oscillations activate NFκB to induce cell migration, whereas high frequency Ca^{2+} oscillations tend to activate NFAT. On the other hand, CREB activation occurs regardless of Ca^{2+} oscillations (Lai et al., 2021). In this study, we found that illumination triggers Ca^{2+} oscillation by phosphorylation at the Ser616 residue but not dephosphorylation at the Ser637 residue of DRP1, via the activation of Ca^{2+}-dependent kinases, CaMKII, ERK, and CDK1 (Figs 3F,G and 4D–I). However, ionomycin has no effect on phosphorylation of DRP1 at Ser616 and dephosphorylation at Ser637 (Fig. 3F,G). Therefore, we propose that optogenetic-engineering plays a critical role in Ca^{2+} oscillation but not steady-state Ca^{2+} by ionomycin. To address the reader's concerns about this data, we will discuss and add a paragraph about "ionomycin has no effect on Ca^{2+} oscillation" to the discussion section.

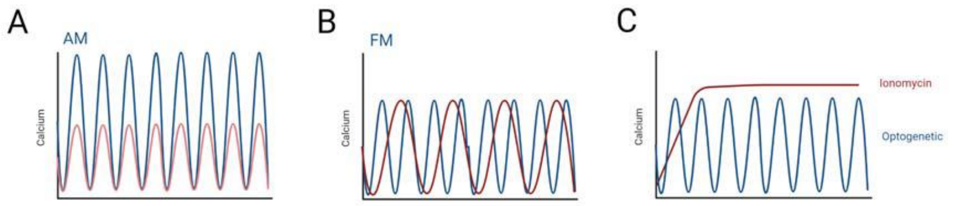

Rebuttal Figure 1. **Different modulations of Ca^{2+} oscillation.** (A) Amplitude modulation, (B) Frequency modulation of Ca^{2+} oscillations. (C) Optogenetic- engineered and ionomycin-induced Ca^{2+} signals.

Reference:
Lai, Y. S., Chang, Y. H., Chen, Y. Y., Xu, J., Yu, C. S., Chang, S. J., Chen, P. S., Tsai, S. J. and Chiu, W. T. (2021). Ca^{2+}-regulated cell migration revealed by optogenetically engineered Ca^{2+} oscillations. *J. Cell. Physiol.* **236**, 4681-4693.
Palk, L., Sneyd, J., Patterson, K., Shuttleworth, T. J., Yule, D. I., Maclaren, O. and Crampin, E. J. (2012). Modelling the effects of calcium waves and oscillations on saliva secretion. *J. Theor. Biol.* **305**, 45-53.
Samanta, K. and Parekh, A. B. (2017). Spatial Ca^{2+} profiling: decrypting the universal cytosolic Ca^{2+} oscillation. *J. Physiol.* **595**, 3053-3062.

*We have added this part in (**page 19 line 10 to line 11**) to the "Discussion" section of the revised manuscript.*

Ionomycin is an effective Ca^{2+} ionophore that is commonly used to modify intracellular Ca^{2+} concentrations. It can increase and sustain Ca^{2+} concentration, called "steady-state Ca^{2+}" (leaving the initial peak Ca^{2+} response unaffected, but not Ca^{2+} oscillation. In this study, we found that illumination triggered Ca^{2+} oscillation by phosphorylation at the Ser616 residue but not dephosphorylation at the Ser637 residue of DRP1, via the activation of Ca^{2+}-dependent kinases, CaMKII, ERK, and CDK1 (Figs 3F,G and 4D–I). However, ionomycin has no effect on phosphorylation of DRP1 at Ser616 and dephosphorylation at Ser637 (Fig. 3F,G). Therefore, we propose that optogenetic-engineering plays a critical role in Ca^{2+} oscillation but not steady-state Ca^{2+} by ionomycin.

4. Figure 4: For clarity, please state what the inhibitors inhibit.

Response:

Thank you very much for your professional comment. In our manuscript, we did not clearly explain the function of KN-93, U0126, RO-3306, or CsA, nor did we provide functional information

© 2023. Published by The Company of Biologists under the terms of the Creative Commons Attribution License (https://creativecommons.org/licenses/by/4.0/).

in figures or figure legends. We have added information about these inhibitors in (*page 13 line 2 to line 4*) of manuscript, figures 4, 5, 6 and figure legends.

5. Figure 7: H and I show increase in LC3 puncta, blocked by 3-MA. The authors mention mitophagy here and p23 line 19, but they are not specifically detecting mitophagy in these experiments. Mitophagy experiments should be included (e.g. using mito-Keima, mito-SRAI, Parkin translocation to mitochondria and/or PINK1- dependent phosphorylation of ubiquitin at Serine 65 as dedicated readouts of mitophagy), or the text should be clarified to reflect what is being shown in the experiments.

Response:

Thank you very much for the professional opinion. Autophagy is a self- degradative process that includes five steps: initiation, elongation, maturation, fusion and degradation. It cleans up damaged organelles, including mitochondria, endoplasmic reticulum, and peroxisomes, and misfolded or aggregated proteins, and gets rid of intracellular pathogens.

Initiation of the phagophore formation occurs after the ULK1/2 complex induction. Elongation of the phagophore is aided by the ATG12–ATG5-ATG16L1 complex, the class III PtdIns3K complex, LC3-II, and ATG9. Eventually, the expanding membrane closes around its cargo to form an autophagosome, and LC3-II is cleaved from the outer membrane of this structure. The outer membrane of the autophagosome will then fuse with the lysosomal membrane to form an autolysosome (Parzych and Klionsky, 2014). Mitophagy is the selective degradation of defective mitochondria via autophagy. Mitophagy is triggered by cues that signal the mitochondrial damage such as disturbances in mitochondrial fission-fusion dynamics, mitochondrial membrane depolarization, enhanced ROS production, mtDNA damage as well as developmental cues such as erythrocyte maturation, removal of paternal mitochondria (Zhang, 2013; Katayama et al., 2020). As shown in Fig. 7A-H, we found that Ca^{2+} signaling caused by high-frequency light illumination can lead to the loss of mitochondrial ATP production, mitochondrial ROS production increase, and mitochondrial membrane potential decrease. Furthermore, LC3 is widely used as an autophagy marker in assays that measure autophagic activity. When autophagy happens, LC3 is lapidated to turn to LC3-II, associating with the newly generated autophagosome membrane. Therefore, we performed the CatCh-Venus-overexpressing U2OS cells under 6, 12, or 24 h of incubation after 470 nm illumination (0.3 mW/mm^2, 0.1 Hz, 250 ms exposure time) for 30 min. The class III PI3K inhibitor, 3-methyladenine (3-MA), is commonly used to selectively block autophagy.

We found that light-induced Ca^{2+} oscillation significantly increased the puncta of LC3 positive cells, and illumination significantly increased autophagy induction after 6 h, reaching 95% after 12 h (Fig. 7J). In contrast, pretreatment of the cells with 3-MA significantly reduced light-induced autophagy. However, our study did not provide sufficient specific markers (mito-Keima, mito-SRAI, Parkin translocation to mitochondria and/or PINK1-dependent phosphorylation of ubiquitin at Serine 65 as dedicated readouts of mitophagy) for detecting mitophagy to prove that light-induced Ca^{2+} oscillations cause mitophagy. We appreciate your suggestion. It should be clarified that light-induced Ca^{2+} oscillations cause autophagy instead of mitophagy. Therefore, we will change the entirety of the claim "mitophagy" to "autophagy" in our revised manuscript.

<u>References:</u>

Katayama, H., Hama, H., Nagasawa, K., Kurokawa, H., Sugiyama, M., Ando, R., Funata, M., Yoshida, N., Homma, M., Nishimura, T. et al. (2020). Visualizing and modulating mitophagy for therapeutic studies of neurodegeneration. *Cell* **181**, 1176-1187.
Parzych, K. R. and Klionsky, D. J. (2014). An overview of autophagy: morphology, mechanism, and regulation. *Antioxid. Redox Signal.* **20**, 460-473.
Zhang J. (2013). Autophagy and mitophagy in cellular damage control. *Redox Biol.* **1**, 19-23.

6. The authors report that their optogenetic method has increased spatial control, but I didn't see experiments that demonstrate this. For example, can it be used to selectively stimulate specific cells? If not, then the claim of increased spatial control should be omitted.

© 2023. Published by The Company of Biologists under the terms of the Creative Commons Attribution License (https://creativecommons.org/licenses/by/4.0/).

Response:
Thank you for your professional suggestion. Optogenetics allow us to use light to control cellular activity with temporal and spatial precision. In our study, we controlled frequency, amplitude, duty cycle, and work duration to ensure that optogenetically-driven Ca^{2+} oscillations could promote mitochondrial fission by increasing phosphorylation at Ser616 of DRP1 via CaMKII, ERK, and CDK1, but not by altering phosphorylation at S637 of DRP1. However, our study did not demonstrate the ability of optogenetics in the spatial regulation of Ca^{2+} oscillation. We truly appreciate your advice to make our manuscript more precise. Therefore, we removed all of the descriptions about spatial resolution from the "Abstract" section of this manuscript.

Additional minor comments
1. Some typographical/grammatical errors to be corrected
e.g. p11 lines 4-5, 8-9
p14, lines 10-13
p32, line 3
Response:
We sincerely appreciate the information and advice you have shared. We double-checked the grammatical errors that you mention and further checked the whole of our manuscript by a native English speaker. We labeled the typographical and grammatical errors in the marked-up copy and edited them in the revised manuscript.

2. Please could all individual data points be shown in graphs to give a better idea of data spread, and actual p-values given in figure legends.
Response:
Thank you for providing us with constructive advice. We read and checked the requirements for preparing the figures. We have added and shown all individual data points in every quantification. In addition, we presented the actual *P*-values in figures and figure legends.

3. The display of microscopy images could be improved by using colour-blind accessible colours.
Response:
Thank you for your consideration and suggestions. Our fluorescence microscopy images were a little bit small and hard to define in terms of the difference between fission and fusion of mitochondria. Therefore, we revised our figures using enlarged representative microscopy images. As an explanation of the mitochondrial states shown in Fig. 1E, we used the analytic tool MicroP to classify mitochondria into six groups based on their radius and length. Thus, our following figures were defined in the same way as Fig. 1E (Figs. 3B, S1D, S2B and S3B). Furthermore, we used pie charts as shown in Fig. S7 to assist the reader in easily observing the result as demonstrated.

We have added this figure as "Fig. S7" in the supplementary information.

© 2023. Published by The Company of Biologists under the terms of the Creative Commons Attribution License (https://creativecommons.org/licenses/by/4.0/).

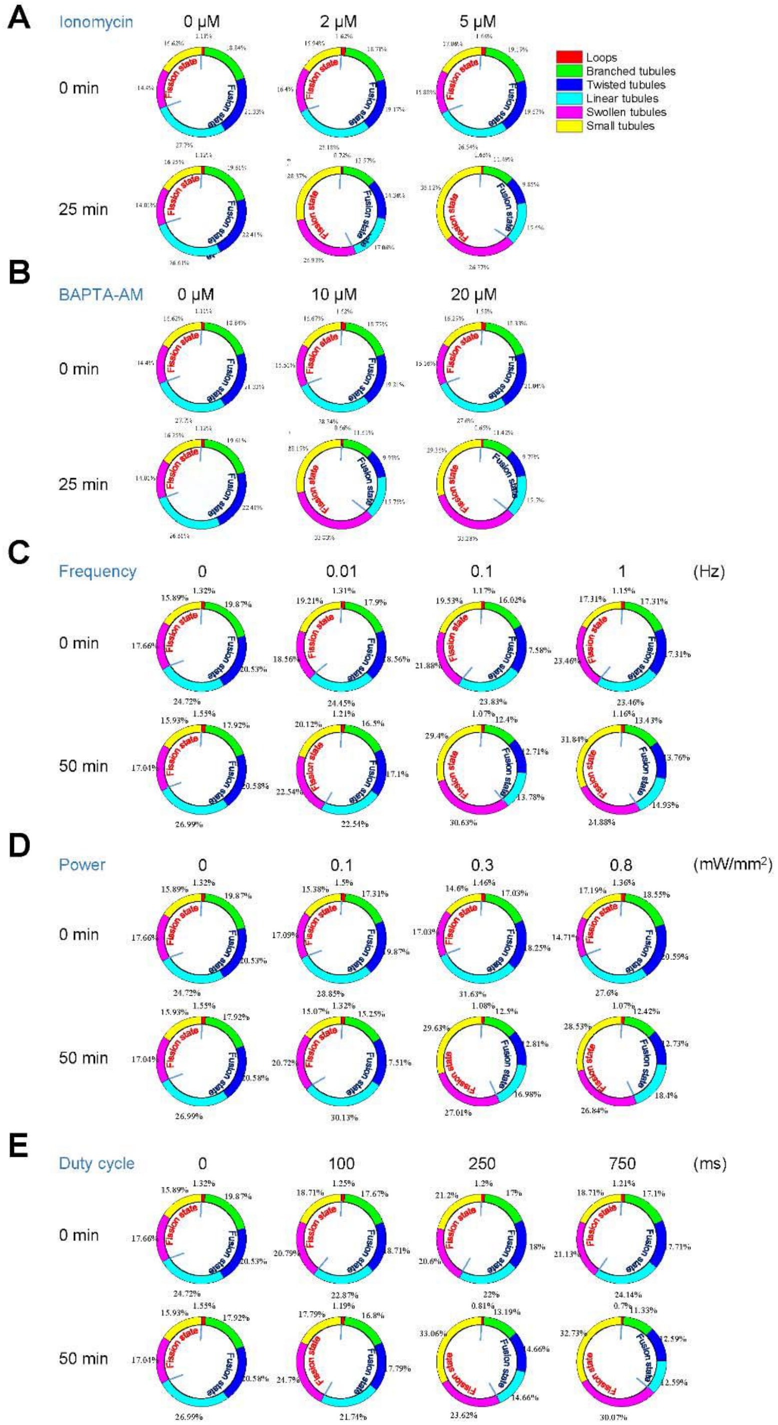

FIGURE S7

Fig. S7. Percentage of six different mitochondrial states using pie chart.

(A) Cytochrome c oxidase subunit 8A (COX8A)-mRFP overexpression in U2OS cells under ionomycin (0, 2, 5 μM) treatment. Quantitative analysis of six different mitochondrial states at the initial point (0 min) and end point (25 min) is presented as a percentage. (B) COX8A-mRFP overexpression in U2OS cells under BAPTA-AM (0, 10, 20 μM) treatment. Quantitative analysis of six different mitochondrial states at the initial point (0 min) and end point (25 min) is presented as a percentage. (C) The CatCh- Venus and COX8A-mRFP-overexpressing U2OS cells were subjected to 470 nm illumination (0.3 mW/mm^2, 250 ms exposure time) at different frequencies (0, 0.01, 0.1, and 1 Hz). Quantitative analysis of six different mitochondrial states at the initial point (0 min) and end point (50 min) is presented as a percentage. (D) The CatCh-Venus and COX8A-mRFP-overexpressing U2OS cells were subjected to 470 nm illumination (0.1

© 2023. Published by The Company of Biologists under the terms of the Creative Commons Attribution License (https://creativecommons.org/licenses/by/4.0/).

Hz, 250 ms exposure time) at different power intensities (0, 0.1, 0.3, and 0.8 mW/mm^2). Quantitative analysis of six different mitochondrial states at the initial point (0 min) and end point (50 min) is presented as a percentage. (E) The CatCh-Venus and COX8A- mRFP-overexpressing U2OS cells were subjected to 470 nm illumination (0.3 mW/mm^2, 0.1 Hz) at different exposure times (0, 100, 250, and 750 ms). Quantitative analysis of six different mitochondrial states at the initial point (0 min) and end point (50 min) is presented as a percentage. MicroP software was used to quantify the whole set of data above.

We thank the reviewer for his/her significant time reviewing this manuscript to provide valuable suggestions and comments. The additional modifications have been provided as shown in the revised manuscript.

Reviewer #2 (Comments to the Author):

This paper provides the useful tool to study the effects of calcium oscillations on mitochondrial function and morphology. Taking into account the importance of calcium signaling in the cell function this methodology is of potential significance to the field. Manuscript is well-written and experimental design is good. I have several suggestions:

1. The inclusion of the ionomycin part is not necessary it doesn't seem to add to the main topic of the study.

Response:
We truly appreciate your consideration. Your opinion appears to be the same as "Question 3" from reviewer#1. We would like to address your question about “why we showed the effect of ionomycin on DRP1 even though it had no effect”. Ca^{2+} signaling is one of the most versatile and universal signaling agents to convert extracellular and intracellular information into specific cellular outcomes, including proliferation, differentiation, muscle contraction, learning and cell death. The interpretation of an increase in intracellular Ca^{2+} signal requires consideration of different properties, such as amplitude, mean concentration, frequency and also the wave speed as mechanisms for signaling (Palk et al., 2012). The cytosolic Ca^{2+} concentration can start oscillating as a result of the stimulation or complex coordinated activity involving many Ca^{2+} transporters in the cell. Ca^{2+} oscillation occurs through amplitude modulation (AM) and frequency modulation (FM) (Samanta and Parekh, 2017). Indeed, optogenetics-induced Ca^{2+} signal belongs to Ca^{2+} oscillations.

In our results, increasing Ca^{2+} concentrations induced a response in the AM of Ca^{2+} oscillations (**Rebuttal Fig. 1A**). In contrast, Ca^{2+} oscillations are decoded by FM, which responds to change in oscillation frequency but not Ca^{2+} concentration (**Rebuttal Fig. 1B**). In addition, different frequencies of intracellular Ca^{2+} oscillating waves seem to be the most common strategy used by the cell to transmit Ca^{2+} messages under many different physiological stimuli (Lai et al., 2021).

Ionomycin is an effective Ca^{2+} ionophore that is commonly used to modify intracellular Ca^{2+} concentrations. It can increase and sustain Ca^{2+} concentration, called "steady-state Ca^{2+}" (leaving the initial peak Ca^{2+} response unaffected), but not Ca^{2+} oscillation (**Rebuttal Fig. 1C**). In our previous study, we found that low frequency Ca^{2+} oscillations activate NFκB to induce cell migration, whereas high frequency Ca^{2+} oscillations tend to activate NFAT. On the other hand, CREB activation occurs regardless of Ca^{2+} oscillations (Lai et al., 2021). In this study, we found that illumination triggers Ca^{2+} oscillation by phosphorylation at the Ser616 residue but not dephosphorylation at the Ser637 residue of DRP1, via the activation of Ca^{2+}-dependent kinases, CaMKII, ERK, and CDK1 (Figs 3F,G and 4D–I). However, ionomycin has no effect on phosphorylation of DRP1 at Ser616 and

© 2023. Published by The Company of Biologists under the terms of the Creative Commons Attribution License (https://creativecommons.org/licenses/by/4.0/).

dephosphorylation at Ser637 (Fig. 3F,G). Therefore, we propose that optogenetic-engineering plays a critical role in Ca^{2+} oscillation but not steady-state Ca^{2+} by ionomycin. To address the reader's concerns about this data, we will discuss and add a paragraph about "ionomycin has no effect on Ca^{2+} oscillation" to the discussion section.

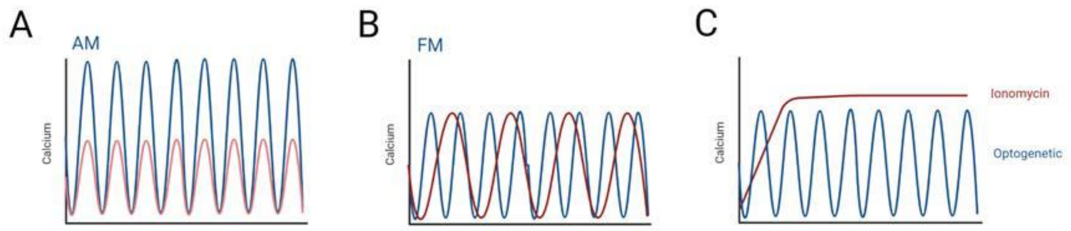

Rebuttal Figure 1. **Different modulations of Ca^{2+} oscillation.** (A) Amplitude modulation, (B) Frequency modulation of Ca^{2+} oscillations. (C) Optogenetic- engineered and ionomycin-induced Ca^{2+} signals.

Reference:

Lai, Y. S., Chang, Y. H., Chen, Y. Y., Xu, J., Yu, C. S., Chang, S. J., Chen, P. S., Tsai, S. J. and Chiu, W. T. (2021). Ca^{2+}-regulated cell migration revealed by optogenetically engineered Ca^{2+} oscillations. *J. Cell. Physiol.* **236**, 4681-4693.

Palk, L., Sneyd, J., Patterson, K., Shuttleworth, T. J., Yule, D. I., Maclaren, O. and Crampin, E. J. (2012). Modelling the effects of calcium waves and oscillations on saliva secretion. *J. Theor. Biol.* **305**, 45-53.

Samanta, K. and Parekh, A. B. (2017). Spatial Ca^{2+} profiling: decrypting the universal cytosolic Ca^{2+} oscillation. *J. Physiol.* **595**, 3053-3062.

*We have added this part in (**page 19 line 10 to line 11**) to the "Discussion" section of the revised manuscript.*

Ionomycin is an effective Ca^{2+} ionophore that is commonly used to modify intracellular Ca^{2+} concentrations. It can increase and sustain Ca^{2+} concentration, called "steady-state Ca^{2+}" (leaving the initial peak Ca^{2+} response unaffected but not Ca^{2+} oscillation. In this study, we found that illumination triggered Ca^{2+} oscillation by phosphorylation at the Ser616 residue but not dephosphorylation at the Ser637 residue of DRP1, via the activation of Ca^{2+}-dependent kinases, CaMKII, ERK, and CDK1 (Figs 3F,G and 4D–I). However, ionomycin has no effect on phosphorylation of DRP1 at Ser616 and dephosphorylation at Ser637 (Fig. 3F,G). Therefore, we propose that optogenetic-engineering plays a critical role in Ca^{2+} oscillation but not steady-state Ca^{2+} by ionomycin.

2. I assume that CsA tests are for exploration of the role of mPTP. This is not stated explicitly. Some more explanations/discussion is needed.

Response:

Thank you for your consideration. The dynamics of mitochondria are not only different in terms of the size or shape of various cell types, but also its rapid morphological remodeling in response to the adaptation of the cell during environmental changes. Mitochondrial morphological changes regulate cell cycle, differentiation, and death via mitochondrial fission and fusion (Karbowski and Youle, 2003; Antico Arciuch et al., 2012; Seo et al., 2018). In particular, the mitochondrial

© 2023. Published by The Company of Biologists under the terms of the Creative Commons Attribution License (https://creativecommons.org/licenses/by/4.0/).

morphological changes are closely involved in overall cellular Ca^{2+} homeostasis (Kowaltowski, et al., 2019).

Dynamin-related protein 1 (DRP1) is responsible for regulating mitochondrial fission and is recruited to the mitochondrial outer membrane and bound by anchored receptors (Smirnova et al., 2001; Loson et al., 2013). In normal condition, DRP1 is commonly located in cytoplasm, and it translocates to the mitochondrial surface and cleavages mitochondrial membrane to facilitate mitochondrial fission (Fonseca et al., 2019). Many recent studies have found that DRP1-mediated mitochondrial fission causes changes in Ca^{2+} dynamics (Favaro et al., 2019; King et al., 2021). Furthermore, DRP1-dependent mitochondrial fission is regulated by phosphorylation at two different conserved sites, serine 616 and 637. These two sites have opposite effects on mitochondrial morphology: phosphorylation of S616 by Cdk1 promotes mitochondrial fission, whereas dephosphorylation of S637 by the Ca^{2+}-dependent phosphatase calcineurin promotes mitochondrial fission. An early study discovered that increased cytosolic Ca^{2+} causes mitochondrial depolarization, which activates the cytosolic phosphatase calcineurin-dependent dephosphorylation of DRP1 S637 (Cereghetti et al., 2008). On the other hand, another study identified the effect of DRP1-mediated Ca^{2+} in different pathways - activation by p-ERK1/2-mediated phosphorylation at S616 or CsA-mediated dephosphorylation at S637 (Wu et al., 2021).

The mitochondrial permeability transition pore (mPTP) is a channel on the mitochondrial inner membrane, composed of cyclophilin D (CypD), adenine nucleotide translocator (ANT) and other substances (Karch and Molkentin, 2014). When cells are subjected to external stress or mitochondrial oxidative stress, mPTP is turned on to cause Ca^{2+} to flow out and eventually lead to cell death (Javadov et al., 2009; Kovac et al., 2017). Overopening of mPTP results in mitochondrial extension due to high osmotic pressure in the matrix, which leads to mitochondrial outer membrane disruption and fragmentation (Zhou et al., 2017). Cyclosporin A (CsA) has also been pointed out as an inhibitor of CypD, and the inhibition of CypD further inhibits the opening of mPTP. (Mishra et al., 2019).

Mdivi-1, an inhibitor of DRP1 translocation, inhibits DRP1, leading to reduced mPTP-induced neurotoxicity in mice (Rappold et al., 2014). Recent studies have shown that DRP1 is recruited next to mPTP under hypoxia, resulting in mitochondrial fragmentation and structural damage due to excessive opening of mPTP, leading to cell death. Interestingly, they found CsA prevented mPTP opening without reducing DRP1 mitochondrial translocation, which may not interfere with the process of mitochondrial fission during hypoxia (Duan et al., 2021). Furthermore, a study found that chronic administration of isoproterenol (ISO) persistently enhanced the frequency of mPTP openings, followed by mitochondrial damage and cardiac dysfunction, which were mediated by the phosphorylation of DRP1-S616 by Ca^{2+}/CaMKII (Xu et al., 2016). These studies demonstrate that even CsA plays an important role in mPTP opening. However, DRP1 acts upstream to mPTP, which is an essential factor for mitochondrial fission. In this study, we investigated the effect of optogenetic on Ca^{2+} oscillation. Therefore, we assumed that the calcineurin signaling pathway may be involved in DRP1-mediated mitochondria fission. Thus, we pre-treated the cells with CsA as a calcineurin inhibitor under light stimulation. However, we did not observe CsA blocking Ca^{2+}-mediated mitochondrial fission at dephosphorylation DRP1-S637. Interestingly, mitochondrial fission induced by optogenetic treatment is related to DRP1-S616 via ERK, CaMKII, and CDK1, but has no impact on DRP1-S637.

References:

Antico Arciuch, V. G., Elguero, M. E., Poderoso, J. J. and Carreras, M. C. (2012). 1150-1180.
Cereghetti, G. M., Stangherlin, A., Martins de Brito, O., Chang, C. R., Blackstone, C., Bernardi, P. and Scorrano, L. (2008). Dephosphorylation by calcineurin regulates translocation of Drp1 to mitochondria. *Proc. Natl. Acad. Sci. U.S.A.* **105**, 15803-15808.
Duan, C., Kuang, L., Hong, C., Xiang, X., Liu, J., Li, Q., Peng, X., Zhou, Y., Wang, H., Liu, L. et al. (2021). Mitochondrial Drp1 recognizes and induces excessive mPTP opening after hypoxia through BAX-PiC and LRRK2-HK2. *Cell Death Dis.* **12**, 1050.

© 2023. Published by The Company of Biologists under the terms of the Creative Commons Attribution License (https://creativecommons.org/licenses/by/4.0/).

Favaro, G., Romanello, V., Varanita, T., Andrea Desbats, M., Morbidoni, V., Tezze, C., Albiero, M., Canato, M., Gherardi, G., De Stefani, D., et al. (2019). DRP1- mediated mitochondrial shape controls calcium homeostasis and muscle mass. *Nat. Commun.* **10**, 2576.

Fonseca, T. B, Sánchez-Guerrero Á, Milosevic, I. and Raimundo N. (2019). Mitochondrial fission requires DRP1 but not dynamins. *Nature* **570**, E34-E42.

Javadov, S., Karmazyn, M. and Escobales, N. (2009). Mitochondrial permeability transition pore opening as a promising therapeutic target in cardiac diseases. *J. Pharmacol. Exp. Ther.* **330**, 670-678

Karbowski, M. and Youle, R. J. (2003). Dynamics of mitochondrial morphology in healthy cells and during apoptosis. *Cell Death Differ.* **10**, 870-880.

Karch, J. and Molkentin, J. D. (2014). Identifying the components of the elusive mitochondrial permeability transition pore. *Proc. Natl. Acad. Sci. U.S.A.* **111**, 10396-10397.

King, W. T., Axelrod, C. L., Zunica, E. R. M., Noland, R. C., Davuluri, G., Fujioka, H., Tandler, B., Pergola K, Hermann, G. E., Rogers, R. C. et al. (2021). Dynamin- related protein 1 regulates substrate oxidation in skeletal muscle by stabilizing cellular and mitochondrial calcium dynamics. *J. Biol. Chem.* **297**, 101196.

Kovac, S., Dinkova Kostova, A. T., Herrmann, A. M., Melzer, N., Meuth, S. G. and Gorji, A. (2017). Metabolic and homeostatic changes in seizures and acquired epilepsy- mitochondria, calcium dynamics and reactive oxygen species. *Int. J. Mol. Sci.* **18**, 1935.

Kowaltowski, A. J., Menezes-Filho, S. L., Assali, E. A., Gonçalves, I. G., Cabral- Costa, J. V., Abreu, P., Miller, N., Nolasco, P., Laurindo, F. R. M. and Bruni- Shirihai, O. S. (2019). Mitochondrial morphology regulates organellar Ca^{2+} uptake and changes cellular Ca^{2+} homeostasis. *FASEB J.* **33**, 13176-13188.

Loson, O. C., Song, Z., Chen, H., and Chan, D. C. (2013). Fis1, Mff, MiD49, and MiD51 mediate Drp1 recruitment in mitochondrial fission. *Mol. Biol. Cell* **24**, 659-667. **Mishra, J., Davani, A. J., Natarajan, G. K., Kwok, W. M., Stowe, D. F. and Camara, A. K. S.** (2019). Cyclosporin a increases mitochondrial buffering of calcium: an additional mechanism in delaying mitochondrial permeability transition pore opening. *Cells* **8**, 1052.

Rappold, P. M., Cui, M., Grima, J. C., Fan, R. Z., de Mesy-Bentley, K. L., Chen, L., Zhuang, X., Bowers, W. J. and Tieu, K. (2014). Drp1 inhibition attenuates neurotoxicity and dopamine release deficits in vivo. *Nat. Commun.* **5**, 5244.

Seo, B. J., Yoon, S. H. and Do, J. T. (2018). Mitochondrial dynamics in stem cells and differentiation. *Int. J. Mol. Sci.* **19**, 3893.

Smirnova, E., Griparic, L., Shurland, D. L. and van der Bliek, A. M. (2001). Dynamin-related protein Drp1 is required for mitochondrial division in mammalian cells. *Mol. Biol. Cell* **12**, 2245-2256.

Wu, Q. R., Zheng, D. L., Liu, P. M., Yang, H., Li, L. A., Kuang, S. J., Lai, Y. Y., Rao, F., Xue, Y. M., Lin, J. J. et al. (2021). High glucose induces Drp1-mediated mitochondrial fission via the Orai1 calcium channel to participate in diabetic cardiomyocyte hypertrophy. *Cell Death Dis.* **12**, 216.

Xu, S., Wang, P., Zhang, H., Gong, G., Gutierrez Cortes, N., Zhu, W., Yoon, Y., Tian, R. and Wang, W. (2016). CaMKII induces permeability transition through Drp1 phosphorylation during chronic ß-AR stimulation. *Nat. Commun.* **7**, 13189.

Zhou, H., Hu, S., Jin, Q., Shi, C., Zhang, Y., Zhu, P., Ma, Q., Tian, F. and Chen, Y. (2017). Mff-Dependent mitochondrial fission contributes to the pathogenesis of cardiac microvasculature ischemia/reperfusion injury via induction of mROS-mediated cardiolipin oxidation and HK2/VDAC1 disassociation-involved mPTP Opening. *J. Am. Heart Assoc.* **6**, e005328.

*We have added this part in (**page 20 line 16 to line 25 and page 21 line 1 to line 9**) to the "Discussion" section of the revised manuscript.*

Mdivi-1, an inhibitor of DRP1 translocation, inhibits DRP1, leading to reduced mPTP-induced neurotoxicity in mice (Rappold et al., 2014). Recent studies have shown that DRP1 is recruited next to mPTP under hypoxia, resulting in mitochondrial fragmentation and structural damage due to excessive opening of mPTP, leading to cell death. Interestingly, they found CsA prevented mPTP opening without reducing DRP1 mitochondrial translocation, which may not interfere with the process of mitochondrial fission during hypoxia (Duan et al., 2021). Furthermore, a study found that chronic administration of isoproterenol (ISO) persistently enhanced the frequency of mPTP openings, followed by mitochondrial damage and cardiac dysfunction, which were mediated by the

© 2023. Published by The Company of Biologists under the terms of the Creative Commons Attribution License (https://creativecommons.org/licenses/by/4.0/).

phosphorylation of DRP1-S616 by Ca^{2+}/CaMKII (Xu et al., 2016). These studies demonstrate that even CsA plays an important role in mPTP opening. However, DRP1 acts upstream to mPTP, which is an essential factor for mitochondrial fission. In this study, we investigated the effect of optogenetic on Ca^{2+} oscillation. Therefore, we assumed that the calcineurin signaling pathway may be involved in DRP1-mediated mitochondria fission. Thus, we pre-treated the cells with CsA as a calcineurin inhibitor under light stimulation. However, we did not observe CsA blocking Ca^{2+}-mediated mitochondrial fission at dephosphorylation DRP1-S637. Interestingly, mitochondrial fission induced by optogenetic treatment is related to DRP1-S616 via ERK, CaMKII, and CDK1, but has no impact on DRP1-S637.

We have added these references to the "References Section" of the revised manuscript.

Duan, C., Kuang, L., Hong, C., Xiang, X., Liu, J., Li, Q., Peng, X., Zhou, Y., Wang, H., Liu, L. et al. (2021). Mitochondrial Drp1 recognizes and induces excessive mPTP opening after hypoxia through BAX-PiC and LRRK2-HK2. *Cell Death Dis.* **12**, 1050.
Rappold, P. M., Cui, M., Grima, J. C., Fan, R. Z., de Mesy-Bentley, K. L., Chen, L., Zhuang, X., Bowers, W. J. and Tieu, K. (2014). Drp1 inhibition attenuates neurotoxicity and dopamine release deficits in vivo. *Nat. Commun.* **5**, 5244.
Xu, S., Wang, P., Zhang, H., Gong, G., Gutierrez Cortes, N., Zhu, W., Yoon, Y., Tian, R. and Wang, W. (2016). CaMKII induces permeability transition through Drp1 phosphorylation during chronic ß-AR stimulation. *Nat. Commun.* **7**, 13189.

3. Ideally authors should include experiments with TMRM to address the issue of membrane potential changes as JC-1 probe is not optimal for these measurements.
Response:
Thank you for your professional advice. JC-1 is a novel cationic carbocyanine dye that is widely used to detect mitochondrial membrane potential (MMP). When the positively charged reagent enters the cell, it combines with the negative charge on the mitochondrial membrane to dimerize and produce red fluorescence. However, when the MMP activity diminishes, JC-1 dye becomes a monomer and emits green fluorescence in the cytoplasm. In our result, we found that optogenetic treatment decreased MMP at frequency 1 Hz and reduced JC-1 aggregation in the mitochondria. Although JC-1 can demonstrate MMP, there is insufficient evidence to conclude that optogenetics cause mitochondrial dysfunction and cell death. We totally agree with your suggestion. Therefore, we further conducted the MMP detection using TMRM. We performed CatCh-Venus-overexpressing U2OS cells stained with TMRM (50 nM Tetramethylrhodamine, methyl ester (TMRM)) under 470 nm illumination (0.3 mW/mm^2, 250 ms exposure time) at different frequencies (0, 0.01, 0.1, and 1 Hz) for 10, 30, 60 min. As the result shown in Fig. 7H, cells illuminated at high frequency (1 Hz) significantly decreased MMP. Therefore, optogenetically engineered Ca^{2+} oscillation induces defects in the MMP, leading to mitochondrial dysfunction and cell death.

We have added this data as "Fig. 7H" in the revised manuscript.

© 2023. Published by The Company of Biologists under the terms of the Creative Commons Attribution License (https://creativecommons.org/licenses/by/4.0/).

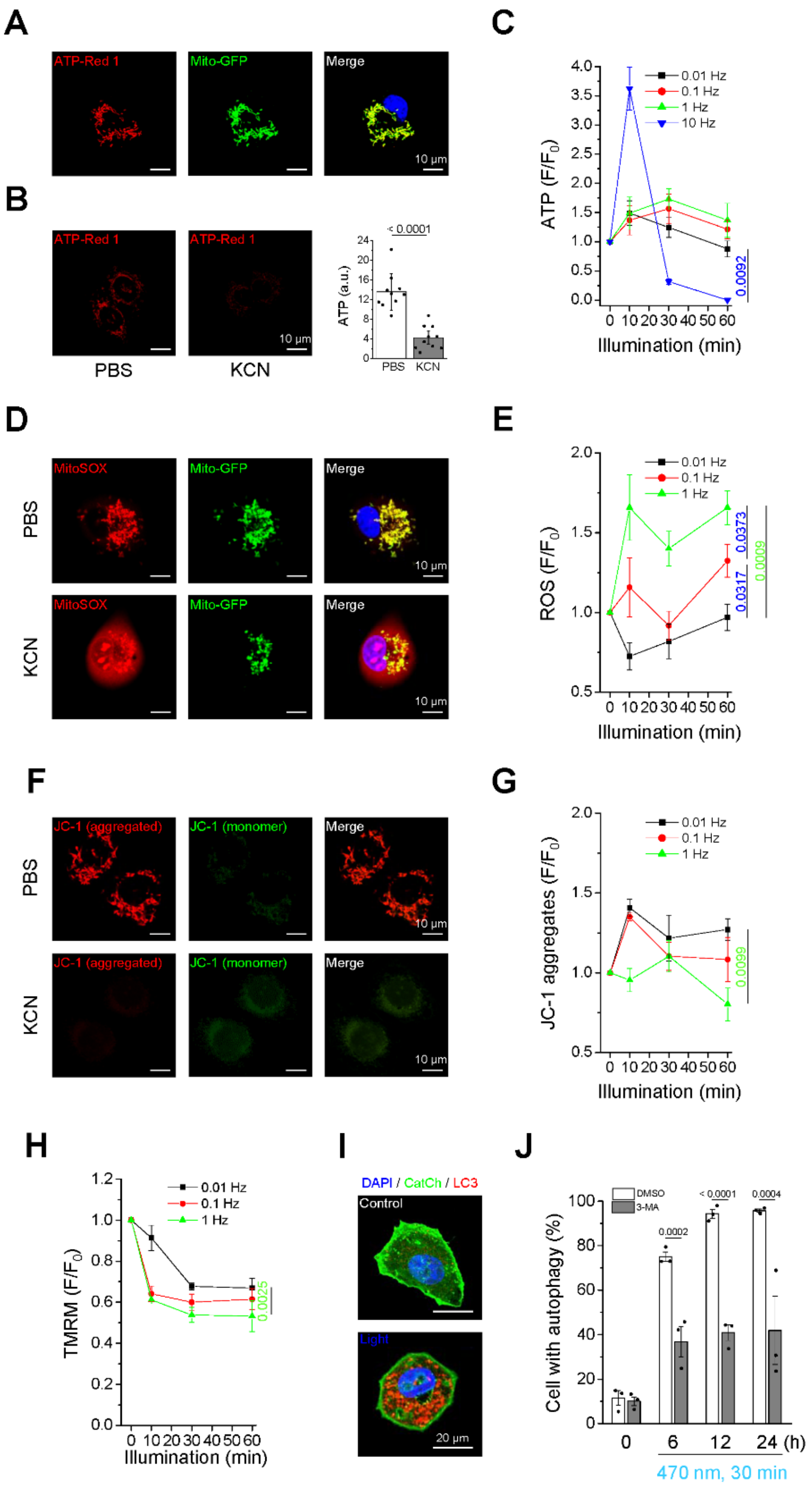

Fig. 7. High frequency Ca^{2+} oscillations induce mitochondrial dysfunction.

© 2023. Published by The Company of Biologists under the terms of the Creative Commons Attribution License (https://creativecommons.org/licenses/by/4.0/).

Real-time fluorescence images showed the dynamic changes in mitochondrial ATP, superoxide levels, and membrane potential. The CatCh-Venus-overexpressing U2OS cells stained with mitochondrial ATP indicator (5 μM ATP-Red 1), superoxide indicator (5 μM MitoSOX), or membrane potential indicator (5 μg/mL JC-1 and 50 nM Tetramethylrhodamine, methyl ester (TMRM)) under treatment with 470 nm illumination (0.3 mW/mm^2, 250 ms exposure time) at different frequencies (0, 0.01, 0.1, and 1 Hz) for 60 min. Representative confocal images of (A,B) ATP-Red 1, (D) (Mito-GFP) when cells were treated with or without KCN for 30 min. Mito-GFP was used as the mitochondrial marker, whereas KCN was used for mitochondrial ATP depletion. Scale bar, 10 μm. (B,C,E,G,H) Quantitative analysis of mitochondrial (B,C) ATP, (E) superoxide levels, and (G,H) membrane potential under 470 nm illumination at different time points using flow cytometry. All values are represented as the mean ± SEM from three biological replicates using one-way ANOVA; adjusted *P*-value is indicated for each comparison. (I) The CatCh-Venus-overexpressing U2OS cells under 6, 12, or 24 h of incubation after 470 nm illumination (0.3 mW/mm^2, 0.1 Hz, 250 ms exposure time) for 30 min. Immunofluorescence staining was performed to label LC3, and the fluorescence images were obtained using confocal microscopy (Scale bar, 20 μm). Moreover, 4',6-diamidino-2-phenylindole dihydrochloride (DAPI) was used as the nucleus marker. (J) Quantitative analysis of the autophagic cells after 470 nm illumination with or without autophagy inhibitor (5 mM 3-MA) pretreatment. All values are represented as the mean ± SEM from three biological replicates using the Student's *t*-test; adjusted *P*-value is indicated for each comparison. KCN, potassium cyanide.

Second decision letter

MS ID#: JOCES/2022/260819

MS TITLE: Optogenetically engineered Ca^{2+} oscillation-mediated DRP1 activation promotes mitochondrial fission and cell death

AUTHORS: Yi-Shyun Lai, Cheng-Chi Chang, Yong-Yi Chen, Thi My Hang Nguyen, Jixuan Xu, Ying-Chi Chen, Yu-Fen Chang, Chia-Yih Wang, Pai-Sheng Chen, Shih-Chieh Lin, I-Chen Peng, Shaw-Jenq Tsai, and Wen-Tai Chiu
ARTICLE TYPE: Research Article

We have now reached a decision on the above manuscript.

To see the reviewers' reports and a copy of this decision letter, please go to: https://submit-jcs.biologists.org and click on the 'Manuscripts with Decisions' queue in the Author Area. (Corresponding author only has access to reviews.)

As you will see, the reviewers gave favourable reports and recommend publication. Reviewer 1 did, however, ask for one sentence to be clarified. It is easier to do it at this stage than at the proofs so I hope you can address this point because I would like to be able to accept your paper once it is returned.

Please ensure that you clearly highlight the change concerning this sentence in the revised manuscript. Please avoid using 'Tracked changes' in Word files as these are lost in PDF conversion.

It is sufficient to mention what you have done in the 'Response to Reviewers' box. in a sentence or two.

Reviewer 1

Advance summary and potential significance to field

As before.

© 2023. Published by The Company of Biologists under the terms of the Creative Commons Attribution License (https://creativecommons.org/licenses/by/4.0/).

Comments for the author

The authors have addressed the points that I previously raised and I would be happy to recommend publication.

My only new additional comment is that the newly edited p.16 line 4 "Autophagy is the selective degradation of defective mitochondria via autophagy, such as the clearance of fragmented mitochondria caused by mitochondrial fission." no longer makes sense, and would need to be rectified.

Reviewer 2

Advance summary and potential significance to field

concerns have been addressed

Comments for the author

concerns have been addressed

Second revision

Author response to reviewers' comments

We thank the reviewer for reviewing this manuscript to provide valuable suggestions and comments. The additional modifications have been provided as shown in the revised manuscript.

Reviewer #1 (Comments for the Author):

The authors have addressed the points that I previously raised and I would be happy to recommend publication.
My only new additional comment is that the newly edited p.16 line 4 "Autophagy is the selective degradation of defective mitochondria via autophagy, such as the clearance of fragmented mitochondria caused by mitochondrial fission." no longer makes sense, and would need to be rectified.

Response:
We appreciate your time and your constructive suggestions for our manuscript. Our study found that light-induced Ca^{2+} oscillation significantly increased the puncta of LC3-positive cells, and illumination significantly increased autophagy induction. As per your suggestion in the previous revision, we have changed the entirety of the claim "mitophagy" to "autophagy" in our revised manuscript. However, we did not link the meaning of the sentence on page 16, line 4, with the sentences in the before and after parts. We deeply thank you for your advice. We will delete this sentence because it no longer makes sense in this result part, and we have added one more sentence to verify why we would like to perform further experiments.

*We have added this sentence to (**page 15 and line 23 to line 25**) of the " Results " section of the revised manuscript .*

Microtubule-associated protein light chain 3 (LC3) is widely used as an autophagy marker in assays that measure autophagic activity.

We thank the reviewer for his/her significant time reviewing this manuscript in order to address outstanding unresolved concerns in this manuscript.

© 2023. Published by The Company of Biologists under the terms of the Creative Commons Attribution License (https://creativecommons.org/licenses/by/4.0/).

Reviewer #2 (Comments for the Author):
Reviewer 2 Advance Summary and Potential Significance to Field: concerns have been addressed

Reviewer 2 Comments for the Author: concerns have been addressed.

Third decision letter

MS ID#: JOCES/2022/260819

MS TITLE: Optogenetically engineered Ca^{2+} oscillation-mediated DRP1 activation promotes mitochondrial fission and cell death

AUTHORS: Yi-Shyun Lai, Cheng-Chi Chang, Yong-Yi Chen, Thi My Hang Nguyen, Jixuan Xu, Ying-Chi Chen, Yu-Fen Chang, Chia-Yih Wang, Pai-Sheng Chen, Shih-Chieh Lin, I-Chen Peng, Shaw-Jenq Tsai, and Wen-Tai Chiu
ARTICLE TYPE: Research Article

I am happy to tell you that your manuscript has been accepted for publication in Journal of Cell Science, pending standard ethics checks.

© 2023. Published by The Company of Biologists under the terms of the Creative Commons Attribution License (https://creativecommons.org/licenses/by/4.0/).
